# Metabolic alterations of peripheral blood immune cells and heterogeneity of neutrophil in intracranial aneurysms patients

**DOI:** 10.1002/ctm2.1572

**Published:** 2024-02-05

**Authors:** Xiaolong Ya, Long Ma, Chenglong Liu, Peicong Ge, Yiqiao Xu, Zhiyao Zheng, Siqi Mou, Rong Wang, Qian Zhang, Xun Ye, Dong Zhang, Yan Zhang, Wenjing Wang, Hao Li, Jizong Zhao

**Affiliations:** ^1^ Department of Neurosurgery Beijing Tiantan Hospital Capital Medical University Beijing China; ^2^ China National Clinical Research Center for Neurological Diseases Beijing China; ^3^ School of Clinical Medicine Capital Medical University Beijing China; ^4^ Department of Neurosurgery Peking Union Medical College Hospital Chinese Academy of Medical Sciences and Peking Union Medical College Beijing China; ^5^ Medical School University of Chinese Academy of Sciences Beijing China; ^6^ Department of Neurosurgery Beijing Hospital Beijing China; ^7^ Beijing Institute of Hepatology Beijing YouAn Hospital Capital Medical University Beijing China

**Keywords:** CyTOF, heterogeneity of neutrophils, immune metabolism, intracranial aneurysms

## Abstract

**Background:**

Intracranial aneurysms (IAs) represent a severe cerebrovascular disease that can potentially lead to subarachnoid haemorrhage. Previous studies have demonstrated the involvement of peripheral immune cells in the formation and progression of IAs. Nevertheless, the impact of metabolic alterations in peripheral immune cells and changes in neutrophil heterogeneity on the occurrence and progression of IAs remains uncertain.

**Methods:**

Single‐cell Cytometry by Time‐of‐Flight (CyTOF) technology was employed to profile the single‐cell atlas of peripheral blood mononuclear cells (PBMCs) and polymorphonuclear cells (PMNs) in 72 patients with IAs. In a matched cohort, metabolic shifts in PBMC subsets of IA patients were investigated by contrasting the expression levels of key metabolic enzymes with their respective counterparts in the healthy control group. Simultaneously, compositional differences in peripheral blood PMNs subsets between the two groups were analysed to explore the impact of altered heterogeneity in neutrophils on the initiation and progression of IAs. Furthermore, integrating immune features based on CyTOF analysis and clinical characteristics, we constructed an aneurysm occurrence model and an aneurysm growth model using the random forest method in conjunction with LASSO regression.

**Results:**

Different subsets exhibited distinct metabolic characteristics. Overall, PBMCs from patients elevated CD98 expression and increased proliferation. Conversely, CD36 was up‐regulated in T cells, B cells and monocytes from the controls but down‐regulated in NK and NKT cells. The comparison also revealed differences in the metabolism and function of specific subsets between the two groups. In terms of PMNs, the neutrophil landscape within patients group revealed a pronounced shift towards heightened complexity. Various neutrophil subsets from the IA group generally exhibited lower expression levels of anti‐inflammatory functional molecules (IL‐4 and IL‐10). By integrating clinical and immune features, the constructed aneurysm occurrence model could precisely identify patients with IAs with high prediction accuracy (AUC = 0.987). Furthermore, the aneurysm growth model also exhibited superiority over ELAPSS scores in predicting aneurysm growth (lower prediction errors and out‐of‐bag errors).

**Conclusion:**

These findings enhanced our understanding of peripheral immune cell participation in aneurysm formation and growth from the perspectives of immune metabolism and neutrophil heterogeneity. Moreover, the predictive model based on CyTOF features holds the potential to aid in diagnosing and monitoring the progression of human IAs.

## Graphical abstract

1. *Immune cell metabolism*: Uncovering metabolic shifts in peripheral immune cells linked to intracranial aneurysm formation.
2. *Neutrophil heterogeneity*: Revealing the roles of distinct peripheral neutrophil subsets in intracranial aneurysm formation.
3. *Predictive model*: The models based on CyTOF features demonstrated excellent performance in predicting intracranial aneurysm occurrence and growth.

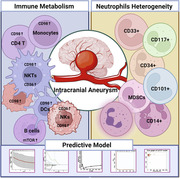

## 1 INTRODUCTION

Intracranial aneurysms (IAs) are acquired lesions, most commonly located at the branching points of intracranial arteries.^1^ The prevalence of IAs in adults ranges from 1 to 2%.^2^ Subarachnoid haemorrhage (SAH) is a severe complication caused by the rupture of aneurysms, with a high mortality and disability rate.^3^ The disruption of the local immune microenvironment, characterised by the infiltration of lymphocytes and macrophages, is commonly considered a significant mechanism in the formation and development of IAs.^4^, ^5^ However, recent studies have highlighted the crucial role played by the peripheral immune environment in these mechanisms.^6^, ^7^

Chronic inflammation, such as periodontitis, might exert sustained disturbances on the peripheral immune environment, potentially altering the pro‐inflammatory activity of specific peripheral blood mononuclear cells (PBMCs) and impacting the formation of IAs.^8^ Relevant high‐dimensional peripheral blood analyses have indicated disproportions and immune activation phenomena in T cells, monocytes and B cells within the peripheral blood of aneurysm patients.^9^ These evidences suggested that PBMCs, as key contributors to the peripheral immune environment, played a significant role in the onset of IAs. In recent years, researchers have discovered that the imbalance in immune cell proportions and functional alterations is closely associated with changes in cellular metabolic activity.^10^, ^11^ When immune cells are activated, they experience substantial shifts in their metabolism to fulfil the energy requirements necessary for their effector functions.^12^, ^13^ This pattern of metabolic reprogramming is a hallmark of immune cell activation and differentiation. Research indicated that immune metabolism abnormalities in PBMCs played a pivotal role in several diseases.^14^ However, it remains unclear whether patients with aneurysms exhibit metabolic abnormalities in PBMCs. Therefore, exploring the immune metabolic changes in PBMCs of IA patients provides valuable insights into the underlying pathological mechanisms of IA, offering new potential targets for the diagnosis and treatment of this condition.

Clinical studies have revealed a close correlation between the elevated neutrophil‐to‐lymphocyte ratio in peripheral blood and the instability and growth of aneurysms.^15^ Other studies have identified polymorphonuclear leukocytes (PMNs) in peripheral blood as crucial circulating mediators influenced by specific gut microbiota, impacting the formation and progression of aneurysms.^16^ These pieces of evidence collectively indicated that PMNs, as another significant component in constructing the peripheral immune environment, played a pivotal role in the progression and rupture of IAs. Transcriptional analysis of peripheral blood PMNs has identified specific transcriptional characteristics within certain neutrophil subsets, which were closely associated with the occurrence of IAs.^17^ Recent researches also have unveiled significant heterogeneity within neutrophils, which were traditionally considered a homogenous population, revealing structural and functional differences among subgroups.^18^ Some neutrophil subsets possessed not only the ability to combat infections and trigger inflammatory responses but also exhibited remarkable anti‐inflammatory capabilities and the potential to promote tissue repair.^19^, ^20^ Aberrant inflammation caused by heterogeneous neutrophils has been closely associated with the development of various chronic inflammatory diseases.^21^ However, it remains unclear whether alterations in the heterogeneity of PMNs are present in the peripheral blood of patients with aneurysms. Hence, investigating neutrophil heterogeneity can also provide deeper insights into the changes in immune responses during the pathogenesis of aneurysms.

CyTOF offers the advantage of high‐dimensional single‐cell analysis, allowing for comprehensive characterisation of cell populations based on numerous parameters simultaneously.^22^ Previous studies have capitalised on these distinctive features of mass cytometry and developed the single‐cell metabolic regulome profiling (scMEP) technology.^10^, ^23^, ^24^ This method employs a high‐dimensional antibody approach to quantifies proteins that regulate metabolic pathway activity. This method allows for a direct comparative analysis of the metabolic states of all immune cell subtypes without the need for pre‐sorting.^25^ Moreover, specific surface markers and functional markers reacting with distinct subgroups have been used to identify different PBMCs and PMNs cell subsets as well as their functional states. In this study, we employed CyTOF technology to comprehensively profile the single‐cell landscape of PBMCs and PMNs. Exploring the peripheral immune environment from the perspectives of immune metabolism in PBMCs and heterogeneity in neutrophils, we aimed to elucidate their impact on the formation and progression of IAs. Furthermore, based on CyTOF analysis findings, we established a disease prediction model for clinical diagnosis and monitoring the progression of IAs.

## 2 MATERIALS AND METHODS

### 2.1 Human specimens

**FIGURE 1:**
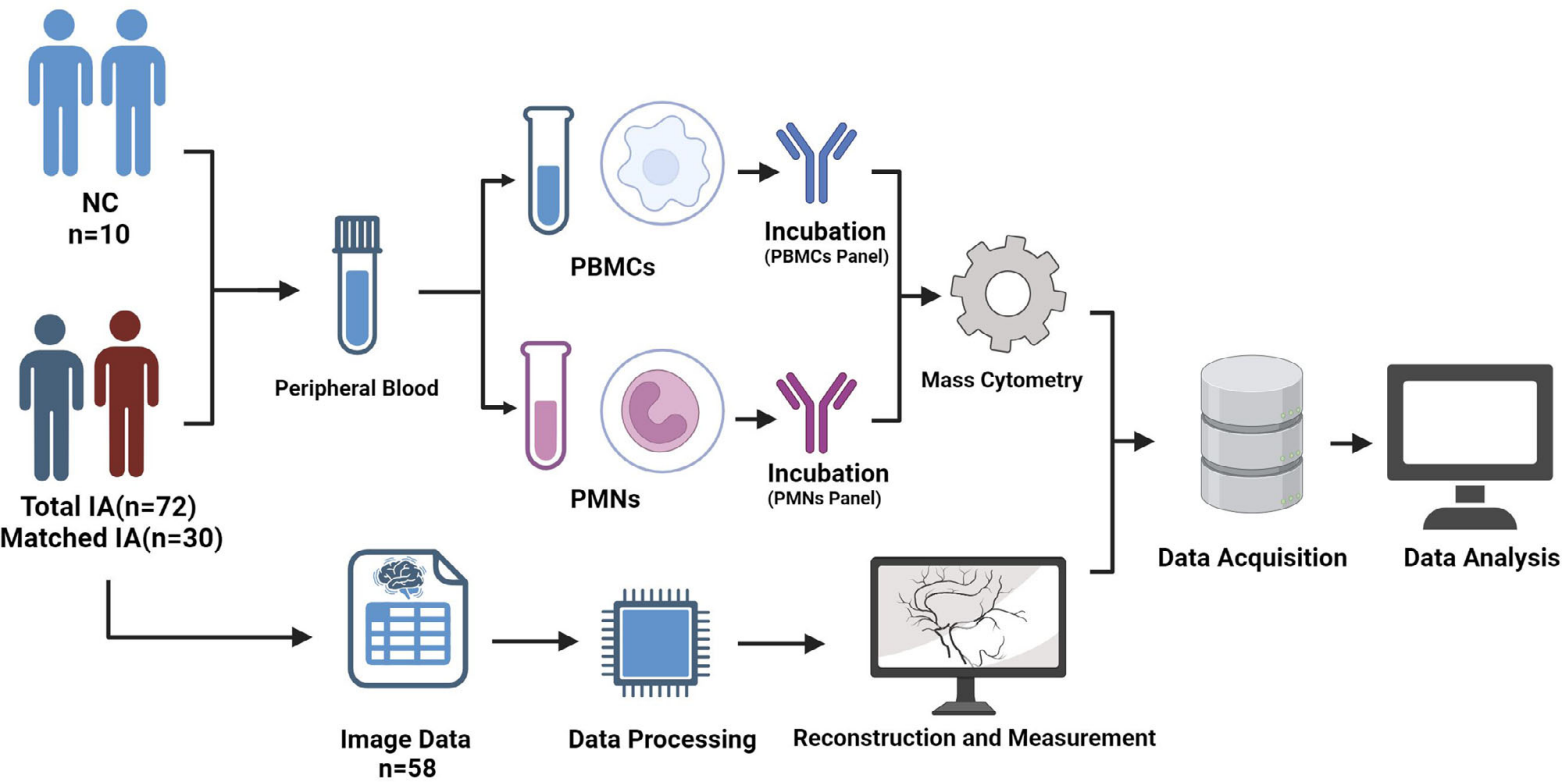
Workflow. Ten healthy controls (NC) and 72 patients with intracranial aneurysm (IA) are enrolled in this study. Peripheral blood samples are collected. PMNs and PBMCs are isolated following centrifugation. After incubation with specific antibodies for PMNs and PBMCs, flow cytometry is performed. CyTOF data from all 72 enrolled patients are employed to illustrate the single‐cell atlas of PBMCs and the single‐cell PMNs profiles. A cohort of 58 patients with complete preoperative imaging data is established for the growth prediction analysis. Following a 1:3 matching principle based on gender and age, a matched cohort (30 matched patients and 10 controls) is established for comparative analysis.

Between October 2022 and April 2023, 72 patients with IAs meeting the inclusion criteria were consecutively enrolled in this study (Inclusion criteria: Patients diagnosed with IAs by DSA, CTA and MRA; Exclusion criteria: (a) Patients with coexisting tumour diseases; (b) Patients with chronic or acute systemic inflammatory diseases; (c) Patients using immunosuppressive medications; (d) Patients who have received chemotherapy, radiotherapy or treatments that may compromise the systemic immune system; (e) Patients with liver or kidney impairment). Peripheral blood samples were collected from patients and healthy controls upon admission to the hospital. Among them, 58 patients had complete raw CTA data available for extracorporeal vascular reconstruction and morphological data measurements. Simultaneously, 10 eligible healthy controls were consecutively recruited (eight with MRA data underwent cerebral vascular examinations and two underwent CTA examinations due to headaches) (Inclusion criteria: (a) Having cerebral vascular imaging data; (b) No IAs confirmed by imaging; Exclusion criteria identical to the aneurysm group). A matched cohort (30 patients with IAs and 10 healthy controls) was created through 1:3 matching based on gender and age for comparative analysis. Detailed clinical information for the 72 patients and 10 healthy volunteers is available in Table S1. Detailed clinical data for the matched cohort can be found in Table S2. The brief experimental workflow of this study is shown in Figure 1.

### 2.2 Isolation and characterisation of single cells from PBMCs and PMNs

Fresh blood samples obtained upon admission were collected in EDTA anticoagulation tubes. Within 2 h, they were layered with 5.0 mL of Polymorphprep (Serumwerk Bernburg AG, 1895). Subsequently, the tubes underwent centrifugation at 500 g for 30 min at 18−22°C. Following centrifugation, the upper band containing PBMCs and the lower band containing PMNs were meticulously aspirated and separately transferred to two centrifuge tubes. These cell fractions were then resuspended in isotonic saline (0.9% NaCl) and subjected to another round of centrifugation.

After centrifugation, cells underwent washing with RBC lysis buffer (Solarbio; R1010) followed by another round of centrifugation. Next, each tube received 0.5 mL of cisplatin (Fluidigm; 201064) for cell viability labelling. The reaction was stopped after a 2‐min incubation by adding 1 mL of 2% FBS (Gibco; 10091−148). After another centrifugation step and removal of the supernatant, cells were fixed with 1 mL of 1.5% paraformaldehyde (Biosharp; BL539A) for 15 min. Neutralisation was achieved with 2 mL of 2% FBS, followed by another centrifugation step. Finally, cells were resuspended in a cell preservation solution (Bioteh; C41100) for subsequent analysis.

### 2.3 Mass cytometry

To assess distinctions among PBMCs, we devised a marker set comprising 36 antibodies (markers of distinguishing various PBMC subsets, markers of metabolic profiles and markers of function). A collection of 24 antibodies (markers of distinguishing various PMN subsets and markers of function) was employed to explore PMN heterogeneity in peripheral blood. These antibodies were procured in a purified state from Biolegend (San Diego, USA) and subsequently labelled in‐house using the Maxpar X8 Multimetal Labeling Kit (Fluidigm, USA), strictly adhering to the instructions. Table S3 (PBMCs panel) and Table S4 (PMNs panel) presented the complete inventory of antibodies employed, along with their corresponding isotopes.

For assessing cell viability, cisplatin‐195Pt (Fluidigm; 201064) was employed as a viability dye. Following a washing step, cell samples were subjected to incubation with cell surface antibodies on ice for 30 min. Subsequently, the samples were permeabilised overnight at 4°C, after which they were stained with intracellular antibodies for 30 min on ice. After thorough washing, the antibody‐labelled samples were incubated with 0.125 nM intercalator‐Ir (Fluidigm) diluted in phosphate‐buffered saline (Sigma–Aldrich, USA) supplemented with 2% formaldehyde. These samples were stored at 4°C until further analysis using mass cytometry. Prior to acquisition, the samples underwent a wash with deionised water and were then resuspended to a concentration of 1 × 106 cells/mL in deionised water containing a 1:20 dilution of EQ Four Element Beads (Fluidigm). Subsequent analysis was performed using the CyTOF2 mass cytometry system (Fluidigm).

### 2.4 CyTOF data analysis

The CyTOF data were acquired in .fcs file format utilising the CyTOF2 system. The incorporation of EQ Four Element Beads was instrumental for normalisation, employing a MATLAB‐based technique. Subsequently, the data were uploaded to Cytobank (https://premium.cytobank.cn). Initially, bead filtering was applied, followed by the application of specific gating criteria to select single cells. Afterward, viable cells were identified based on 193Ir staining and CD45+ cells were gated to isolate PBMCs and PMNs (refer to Figure S1 for details). Further data analysis were performed using the automated dimensionality reduction algorithm, FlowSom, implemented in R. Visualisation of the results was accomplished through tSNE, a visual dimensionality reduction algorithm.

### 2.5 Vascular reconstruction and measurement of morphological parameters

We collected preoperative CTA data in DICOM format from a high‐resolution CTA workstation (Siemens, Berlin, Germany) and converted them into slice DICOM data with a slice thickness of approximately 0.5 mm. Subsequently, the dataset was imported into Mimics 19.0 (Mimics Research 19.0; Materialize, Belgium) and reconstructed for subsequent analysis.

Radiological measurements were conducted by an experienced neurosurgeon (ML) using high‐resolution CTA scans. Measurements of L, d, H, aneurysm angle and aneurysm surface area were obtained from the CTA scans, as illustrated in Figure S2.

### 2.6 Statistical analysis

#### 2.6.1 Baseline analysis

Continuous clinical variables were expressed as mean ± standard deviation, while categorical variables were presented as median and IQR. Chi‐square tests were used for categorical variables, *t*‐tests for normally distributed continuous variables and Wilcoxon tests for non‐normally distributed continuous variables.

#### 2.6.2 The comparison of subsets analysis

Scatter plots were used to visualise the comparison of the proportions of the same subsets between the two groups. The scatter boxplot is used to illustrate the comparative analysis of functional and metabolic molecules. Wilcoxon tests were employed to detect intergroup differences.

#### 2.6.3 Subsets correlation analysis

After normalising the proportions of cell subsets. Pearson correlation coefficients were calculated to assess the relationships between different cell subsets.

#### 2.6.4 Establishment models

We established two predictive models: one for predicting the occurrence of IAs (encompassing 72 enrolled patients and 10 healthy controls) and another for predicting aneurysm growth (focused on 58 enrolled patients with complete radiological data). Initial standardisation procedures were applied to the proportions of immune subsets identified by CyTOF. Randomly selecting 70% of the samples for the training set and the remaining 30% for the test set. LASSO regression was constructed under the condition of *λ* = lambda.min to screen factors (immune cell subsets and clinical features) associated with the occurrence and surface area of aneurysms. Subsequently, the random forest tree model was constructed using the training set.

For the aneurysm occurrence prediction model, we conducted recursive analysis using fivefold cross‐validation using the training set, optimising the model by considering variable weights. Following, the test set is used to validate the predictive model, and model performance was evaluated by the area under the ROC curve.

Regarding the aneurysm growth model, LASSO regression identified a correlation between N03 and the surface area of arterial aneurysms. A growth prediction model was directly constructed using the training set. ELAPSS scores are commonly used in clinical practice to predict the progression of IAs.^26^ In this study, we evaluated the predictive performance of the growth model based on CyTOF immune features in comparison with ELAPSS scores for aneurysm progression using prediction error plots and out‐of‐bag (OBB)*** error plots in the test set.

Statistical significance was defined as a *p* value less than .05. All statistical analyses were conducted using R software.

## 3 RESULTS

### 3.1 The composition of immune cells in PBMCs

**FIGURE 2:**
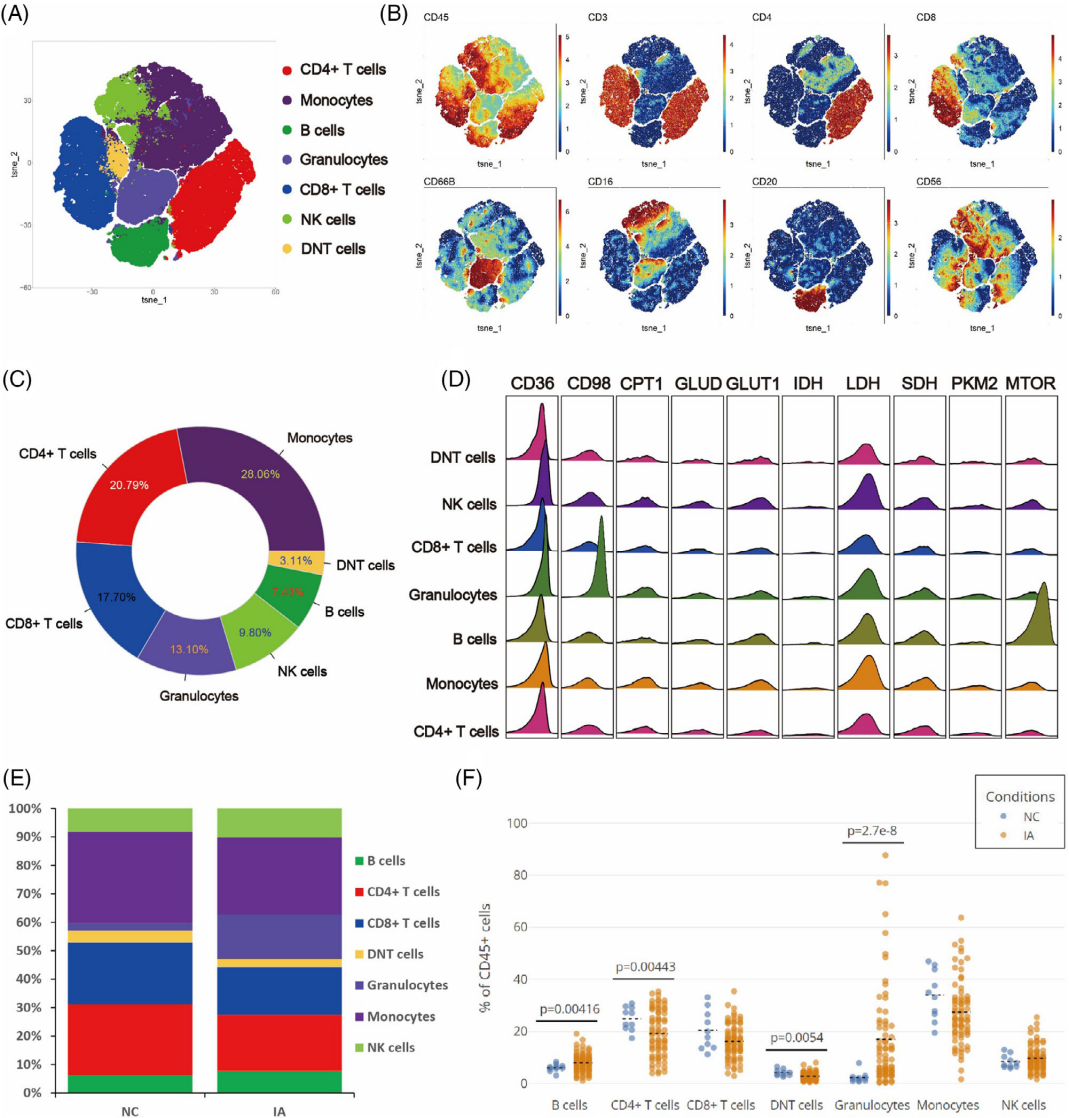
Atlas of PBMCs. The results of PBMCs clustering analysis within the enrolled samples are visualised in the T‐SNE plot (A). Classical markers distinguishing various subpopulations are displayed in a spectral format on the T‐SNE plot (B). A donut chart illustrates the proportions of various subpopulations identified through clustering analysis (numeric values show the proportions and the filled colours correspond to those in the T‐SNE plot, counterclockwise arrangement from high to low at the 3 o'clock position) (C). The ridge plot showcases the expression of metabolic molecules in different subpopulations (D). Stacked bar charts present the relative proportions of each cell subpopulation (filled colours match those in the T‐SNE plot) (E). Scatter plots depict the comparative proportions of various subpopulations between the two groups (each solid circle represents each sample and the black dashed line represents the mean of group).

In order to gain preliminary insights into the composition of immune cells in the peripheral blood of IA patients, an unsupervised Flowsom clustering method was employed to initially analyse the immune cell constitution in peripheral blood samples from 72 IA patients and 10 non‐IA healthy controls (NC). Based on the expression profiles of classical cell surface markers, seven distinct cellular subgroups were identified (Figures 2A and 1B). Among them, monocytes constituted the highest proportion, accounting for 28.06%, whereas DNT cells exhibited the lowest content, representing a mere 3.11% (Figure 2C). Metabolic profiling analysis unveiled the existence of molecules linked with fatty acid metabolism (CD36 and CPT1), amino acid metabolism (CD98 and GLUT1), glucose metabolism‐related entities (GLUD, PKM2 and LDH) as well as molecules implicated in the tricarboxylic acid (TCA) cycle (IDH and SDH) across distinct subsets of cells. Noteworthy, B cells displayed prominent mTOR molecule expression, while CD98 and CD36 molecules were conspicuously expressed in neutrophils (Figure 2D).

Upon comparing the proportions of each subpopulation between the NC and IA groups, we observed that CD4+ T cells (*p* = .00443 < .01) and DNT cells (*p* = .0054 < .01) were predominantly present in the peripheral blood of NC. Conversely, B cells (*p* = .00416 < .01) and neutrophils (*p* < .001) were primarily enriched in the peripheral blood of IA patients (Figures 2E and F).

### 3.2 CD4 T cells derived from the IA group exhibited elevated expression of CD98, coupled with enhanced proliferative capacity and pro‐inflammatory activity

**FIGURE 3:**
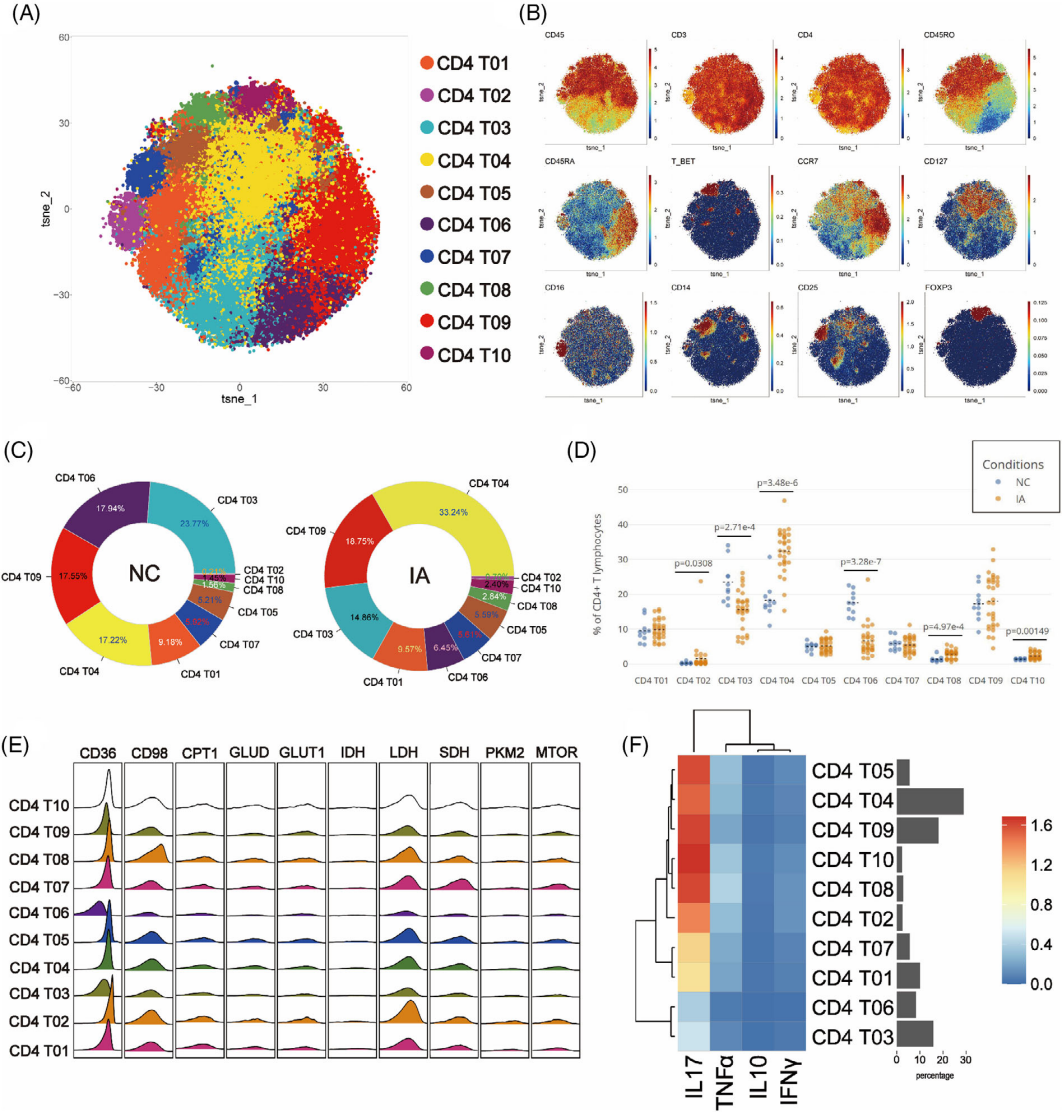
Comparative analysis of CD4+T subsets. The clustering results of CD4 T cell are visualised in the T‐SNE plot, with different colours representing distinct subpopulations (A). Classic markers distinguishing these subpopulations are displayed in a spectral format on the T‐SNE plot (B). Two donut plots illustrate the proportion of cell subpopulations discovered through clustering analysis in the NC and IA groups (numbers represent proportions and the colour filling corresponds to the T‐SNE plot, arranged counterclockwise from high to low starting at 3 o'clock) (C). Scatter plots depict the comparative proportions of each subpopulation between the two groups (each solid circle represents the proportion in each sample, the black dashed line represents the mean of group and statistically significant differences are indicated by displayed *p* values) (D). A ridge plot visualises the metabolic molecule expression in each subpopulation (E). A heatmap displays the expression of functional molecules in each subpopulation (F).

In order to investigate the differences in CD4+ T lymphocytes between IA and NC groups, CD4+ T cells were isolated and subjected to Flowsom clustering analysis. Ten distinct cell subpopulations were identified (Figure 3A). Based on the expression profiles of CD45RA, CD45RO and CCR7, two subgroups of terminal effector memory cells (Temra) (CD4 T09 and CD4 T10), one subgroup of naïve T cells (Tn) (CD4 T06), five subgroups of central memory T cells (Tcm) (CD4 T02, CD4 T04, CD4 T05, CD4 T07 and CD4 T08) and two subgroups of effector memory T cells (Tem) (CD4 T01 and CD4 T03) were annotated (Figure 3B).

In the NC group, Tem predominated (with CD4 T03 exhibiting the highest content, constituting 23.77% of the total CD4 T cell population). Conversely, in the IA group, Tcm predominated (with CD4 T04 being the most abundant, accounting for 33.24%) (Figure 3C). Compared with the CD4 T cell subpopulations in the NC group, there was a decline in the content of Tn (CD4 T06), a subgroup of Tem (CD4 T03) and CD25+ Tcm (CD4 T07) in the IA group, accompanied by an increase in the proportions of other subsets (Figure 3C). Significant statistical differences in the proportions of CD4 T02, CD4 T03, CD4 T04, CD4 T06, CD4 T08 and CD4 T10 were observed between the two groups (Figure 3D).

In various subpopulations, energy supply relied on glycolysis, lipid metabolism and amino acid metabolism. Metabolic analysis revealed consistent patterns, except for lower metabolic activity in Tn (CD4 T06) and a subset of Tem (CD4 T03) (Figures 3E and F). The CD4 T cells came from the NC group mainly expressed higher levels of PKM2 (While statistical significance was not reached in some cell subpopulations, they still exhibited discernible trends), while CD98 expression in CD4 T cells derived from the IA group was higher (The differences among various cell subpopulations between the two groups were all statistically significant.) (Figure S3). These suggested that CD4 T cells derived from the NC group relied more on glucose for energy, while CD4 T cells in IA patients depended more on amino acids. We also observed that CD4 T03 (*p* = .0137 < .05) and CD4 T04 (*p* = .0461 < .05) from the NC group expressed higher levels of mTOR molecules compared to the IA group. Additionally, CD4 T05 (*p* = .0496 < .05) and CD4 T08 (*p* = .0308 < .05) from the NC group exhibited increased CD36 expression. These findings indicated a more intricate metabolic profile within specific cellular subgroups. TCA cycle metabolism showed no significant differences between groups (IDH and SDH had no significant differences.) (Figure S3). Further comparison of the proliferative capacity between two groups revealed that CD4 T cells in the IA group demonstrated higher proliferative capacity overall (Except for CD4 T02 showed a trend of increased Ki67 expression, the rest exhibited statistically significant differences.) (Figure S3). Differences in PD1 expression were only observed in CD4 T01 (*p* < .01) and CD4 T03 (*p* = .026 < .05) (Figure S3).

Enhanced metabolism accompanies heightened functionality. When comparing the expression of functional molecules between two groups, TNF‐α and IL_10 exhibited elevated expression in the IA group (although some did not reach statistical significance, they displayed an increasing trend) (Figure S3). This suggested a more active functional state of CD4 T cells in the IA group. Additionally, we observed that CD4 T02 from IA group expressed higher levels of IL_17 (*p* < .01) and IFNγ (*p* = .0197 < .05), while CD4 T06 expressed more IL_17 in the NC group (*p* = .0137 < .05) (Figure S3). CD4 T01 from IA group exhibited elevated expression of IL_17 (*p* = .0238 < .05) compared with their counterparts in the NC group (Figure S3).

### 3.3 Similar to CD4 T, CD8 T from the IA group exhibited elevated expression of CD98 and enhanced proliferative capacity, accompanied by a decrease in mTOR expression

**FIGURE 4:**
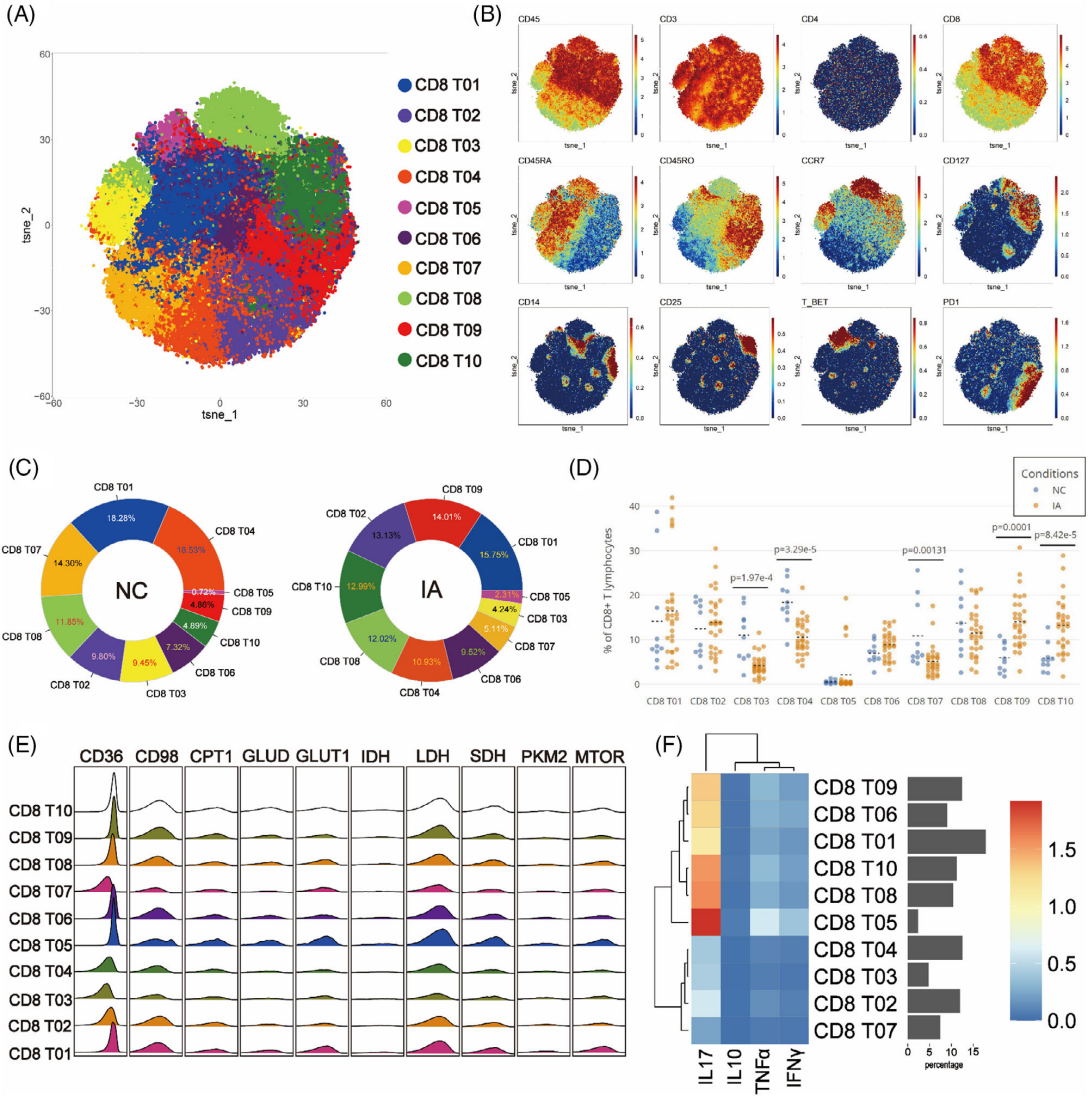
Comparative analysis of CD8+T subsets. The T‐SNE plot (A) visualises the clustering results for CD8 T cells, where various colours signify unique subpopulations. Classic markers differentiate these subpopulations are depicted in spectral format on the T‐SNE plot (B). Two donut plots demonstrate the distribution of cell subpopulations identified by clustering analysis within the NC and IA groups (numbers represent proportions and the colour filling corresponds to the T‐SNE plot, arranged counterclockwise from high to low starting at 3 o'clock) (C). Scatter plots (D) illustrate the relative proportions of each subpopulation between the two groups (each solid circle indicated the proportion in a given sample, the black dashed line signifies the mean of group and statistically significant differences are indicated by displayed *p* values). A ridge plot (E) visually presents the expression of metabolic molecules within each subpopulation. Meanwhile, a heatmap (F) exhibits the expression pattern of functional molecules across each cell subpopulation.

To explore the difference in CD8+ T lymphocytes between the IA and NC groups, CD8+ T cells were isolated and subjected to Flowsom clustering analysis, resulting in the identification of ten distinct cell subpopulations (Figure 4A). Based on the expression profiles of CD45RA, CD45RO and CCR7, one subgroup of Temra (CD8 T08), one subgroup of Tn (CD8 T03), five subgroups of Tcm (CD8 T01, CD8 T05, CD8 T06, CD8 T09 and CD8 T10), two subgroups of Tem (CD4 T02 and CD4 T04) and one subgroup of effective T cells (Teff) (CD8 T07) were annotated (Figure 4B).

In the NC group, CD8 Tem predominated, with CD8 T04 exhibiting the highest proportion at 18.53% of the total CD8 T cell population (Figure 4C). In contrast, within the PBMCs of the IA group, CD8 Tcm predominated, with CD8 T01 being the most abundant, constituting 15.75% (Figure 4C). The IA group showed decreased proportions of CD8 Tn (CD8 T03), CD8 Temra (CD8 T08), CD8 Teff (CD8 T07), a subgroup of Tem (CD8 T04) and Tcm (CD8 T01) compared with the NC group (Figure 4C). Conversely, other subsets exhibited increased. Statistically significant differences were observed in the proportions of CD8 T03, CD8 T04, CD8 T07, CD8 T09 and CD8 T10 between the two groups (Figure 4D).

Glycolysis, lipid metabolism and amino acid metabolism remain pivotal energy sources for CD8 T cells. Metabolic analysis revealed consistent patterns, except for lower metabolic activity in Tn (CD8 T03), Tem (CD8 T04 and CD8 T02) and Teff (CD8 T07) (Figures 4E and F). Similar to CD4 T cells, CD8 T cells from the NC group exhibited elevated expression of PKM2 and CD36 molecules (although some differences among subgroups did not reach statistical significance, they still showed certain trends). Furthermore, CD8 T cell subsets from the NC group consistently exhibited high expression levels of mTOR. (Statistical differences were observed only in CD8 T03 and CD8 T09.) Similar to CD4 T cells, CD8 T cells from the IA group displayed higher expression of CD98 (with statistically significant differences observed across all subsets) (Figure S4). In the comparison, CD8 T05 cells from the NC group exhibited higher expression of the PD1 molecule (*p* = .0498 < .05), whereas conversely, CD8 T10 cells from the IA group showed higher expression of the PD1 molecule (*p* = .00611 < .05) (Figure S4). The comparison of proliferative capacity results remained analogous to CD4 T cells, with all CD8 T cell subgroups from the IA group displaying elevated proliferative capacity (except for CD8 T05, CD8 T06 and CD8 T07, which did not show statistical significance but exhibited a certain trend) (Figure S4).

Functional comparative analysis revealed robust expression of IFNγ, IL‐10 and TNF‐α in various subsets derived from the IA group, underscoring the functional vigour of CD8 T cells within IA PBMCs (Figure S4).

### 3.4 B cells from the IA group showed elevated expression of CD98 and mTOR, accompanied by decreased expression of CD36, GLUD and CPT1

**FIGURE 5:**
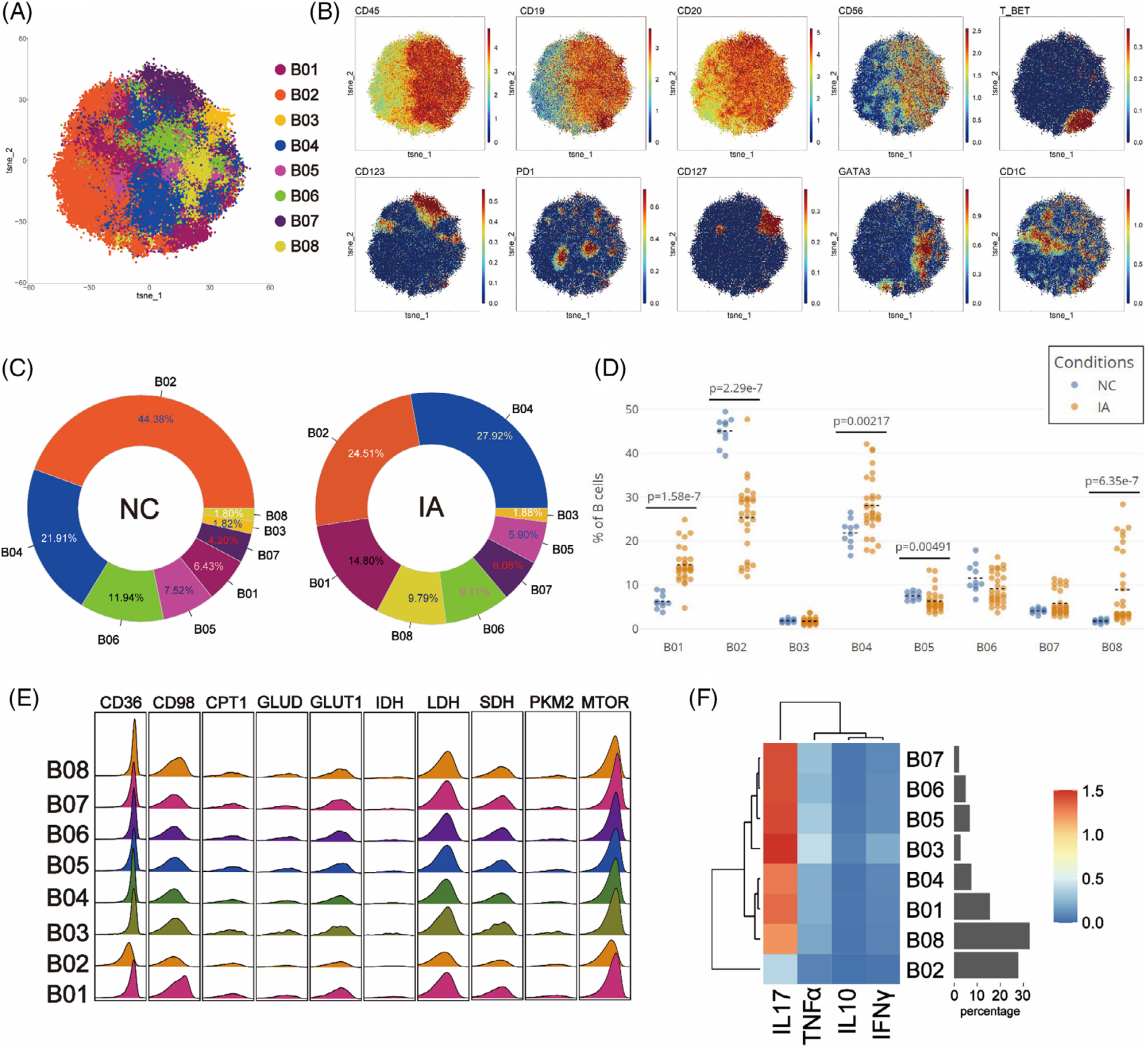
Comparative analysis of B lymphocyte subsets. The T‐SNE plot illustrates the dimensionality reduction of B cell clustering results, where distinct subpopulations are colour coded (A). Classic markers distinguishing these subpopulations are presented in a spectral format on the T‐SNE plot (B). Two donut plots (C) illustrate the proportion of cell subpopulations discovered through clustering analysis in the NC and IA groups (numeric values indicate proportions and colours align with the T‐SNE plot, arranged counterclockwise from high to low starting at 3 o'clock). Scatter plots (D) present a comparison of the proportions of each cell subpopulation between the two groups (each solid circle representing the proportion in a specific sample. The black dashed line signifies the mean of group and statistically significant differences are denoted by displayed *p* values). A ridge plot (E) illustrates the expression of metabolic molecules in each cell subpopulation. Additionally, a heatmap (F) portrays the expression patterns of functional molecules in each cell subpopulation.

To explore potential differences in B cells between the NC and IA groups, B cells were isolated and subjected to Flowsom clustering analysis, resulting in the identification of eight distinct cell subpopulations (Figure 5A). Based on the expression patterns of CD19 and CD20 molecules, the B02 cells were categorised as CD19‐CD20+ subgroups, while the remaining cell subpopulations fell under the CD19+CD20+ category (Figure 5B).

In the PBMCs of the NC group, B02 exhibited the highest abundance (44.38%), while B08 had the lowest representation (1.80%). Conversely, in the IA group, B04 was the most abundant (27.92%), and B03 was the least represented (1.88%) (Figure 5C). Compared with the NC group, there was a decrease in the proportions of B02, B05 and B06, accompanied by an increase in the proportions of other cell subsets within the IA group (Figure 5C). Notably, the differences in proportions of B01, B02, B04, B05 and B08 between the two groups were statistically significant (Figure 5D).

In contrast to T cell metabolic patterns, B cell subsets exhibited higher expression of the mTOR molecule in addition to relying on glycolysis, lipid and protein metabolism for energy (Figure 5E). Furthermore, there was a prevalent elevation in the expression of SDH, a key player in the TCA cycle (Figure 5E). Subgroup comparisons indicated that only B02 may reside in a low metabolic state, in line with its functional characteristics (Figure 5F). B cell subsets from the NC group generally exhibited heightened expression of PKM2. (Only some subsets exhibited statistically significant differences.) Additionally, CD36, GLUD and CPT1 were broadly more expressed across various B cell subsets in the NC group, whereas mTOR and CD98 were generally highly expressed in different B cell subsets from the IA group (although some differences among subsets may not reach statistical significance, the trend persists) (Figure S5). These findings suggested that B cells from the NC group tend to favour a metabolic mode reliant on glycolysis and lipid uptake, while those from the IA group lean towards an amino acid uptake metabolic mode. Except for B05, PD1 expression was higher in all B cell subsets of the NC group compared to the IA group, with only B01 (*p* = .0044 < .01), B04 (*p* = .00246 < .01) and B06 (*p* = .0035 < .01) showing statistical differences (Figure S5). Analysing the proliferative capacity of B cell subsets between the two groups, it was observed that all B cell subsets in the IA group expressed higher levels of KI67 compared with the NC group (except for B02, B04, B07 and B08, where statistically significant differences were observed) (Figure S5). This suggests that B cells from the IA group possess vigorous proliferative capacity.

Functional analysis revealed that B cell subsets from the NC group predominantly expressed IL‐17 and INFγ, whereas B cells from the IA group mainly expressed IL‐10 and TNF‐α (with no significant differences observed among subgroups) (Figure S5).

### 3.5 NKT cells derived from IA exhibited high expression of CD36 and CD98, accompanied by increased proliferative activity. Specific pro‐inflammatory subgroups showed heightened metabolic activity

**FIGURE 6:**
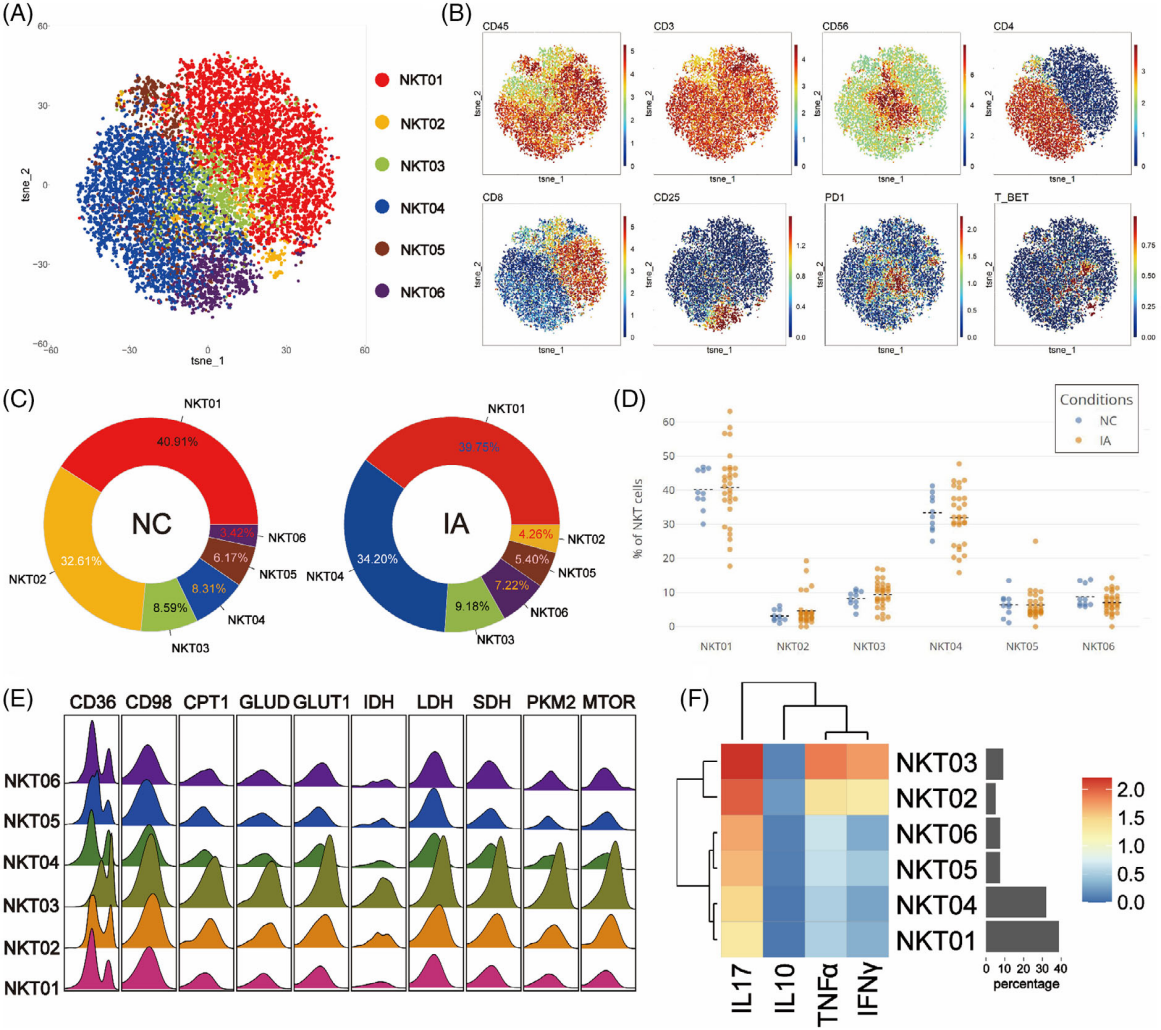
Comparative analysis of NKT subsets. The T‐SNE plot demonstrates the dimensionality reduction of NKT clustering results, with distinct subpopulations colour coded (A). Classic markers distinguishing these subpopulations are displayed in a spectral format on the T‐SNE plot (B). The two donut plots visually represent the proportions of various cell subpopulations identified in the NC and IA groups through clustering analysis (numerical values represent proportions, and colours align with the T‐SNE plot, arranged counterclockwise from high to low starting at 3 o'clock) (C). Scatter plots (D) depict the comparative proportions of each cell subpopulation between the two groups (each solid circle represents the proportion in a specific sample, the black dashed line indicates the mean of group and statistically significant differences are indicated by displayed *p* values). A ridge plot (E) demonstrates the expression of metabolic molecules in each cell subpopulation. Additionally, a heatmap (F) reveals the expression patterns of functional molecules in each cell subpopulation.

To explore potential differences in NKT cell between the NC and IA groups, NKT cells were isolated and subjected to Flowsom dimensionality reduction clustering analysis, resulting in the identification of six distinct cell subsets (Figure 6A). Based on the expression patterns of CD4 and CD8 molecules, NKT05 was categorised as a CD4+CD8+ subgroup, while NKT01 and NKT02 belonged to the CD4−CD8+ subgroup, with NKT02 also expressing T‐bet. NKT04 and NKT06 were classified as CD4+CD8− subgroups, with NKT06 expressing CD25. NKT03 expressed the PD1 molecule (Figure 6B).

Regardless of the NC or IA group, NKT01 was the most abundant subset (NC: 40.91%; IA: 39.75%). NKT02 constituted the smallest proportion in the NC group (3.42%), while in the IA group, NKT02 had the lowest representation (4.26%) (Figure 6C). Comparing subsets between the NC and IA groups, apart from NKT01 and NKT02, the proportions of other subsets increased in the IA group (Figure 6C). The analysis of subgroup proportions revealed that no significant statistical differences were observed between the two groups (Figure 6D).

All metabolic molecules were highly expressed in NKT cell subsets, particularly in NKT03. This suggests that NKT03 was in a heightened metabolic state, which was also reflected in the expression of functional molecules (Figures 6E and F). Comparing metabolic molecule expression of NKT03 between the two groups, GLUT1 and LDH were significantly up‐regulated in the IA group, along with CD36 and GLUD (Figure S6). TCA cycle molecules (IDH and SDH) were also elevated in this subset (Figure S6). Similar patterns were seen in NKT01, with increased CD36, IDH and SDH expression (Figure S6). CD98 molecules remain consistently highly expressed in the IA group, with significant differences (Figure S6). Notably, mTOR expression in NKT04 (*p* = .0334 < .05) and NKT05 (*p* = .0452 < .05) derived from IA group were lower compared to the NC group (Figure S6).

Functional analysis revealed that NKT03 cells from the IA group exhibited higher expression levels of various functional molecules compared to the NC group. Additionally, all NKT cell subsets from the IA group, except NKT02, showed elevated expression of TNF‐α. Moreover, it was observed that NKT02 cells from the IA group exhibited higher expression of IL‐10 (*p* = .0401 < .05), and NKT01 cells showed increased expression of IFNγ (*p* = .026 < .05) (Figure S6).

### 3.6 NK cells derived from IA exhibited high expression of CD98, accompanied by increased proliferative activity. Specific IA‐derived CD56^high^ NK cells showed diverse energy sources

**FIGURE 7:**
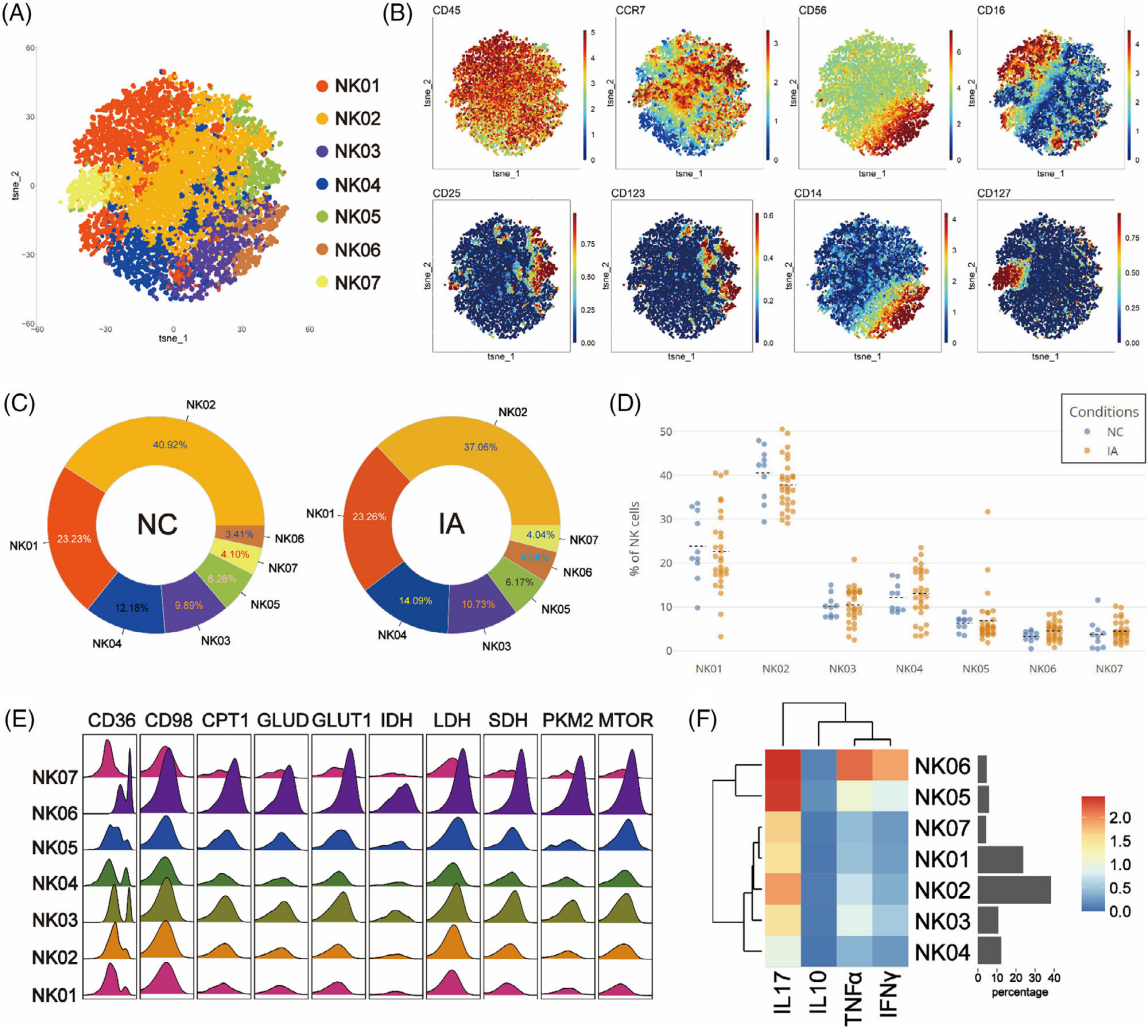
Comparative analysis of NK subsets. The T‐SNE plot showcases the results of NK clustering analysis, where unique subpopulations are colour coded (A). Classic markers distinguishing these subpopulations are displayed in a spectral format on the T‐SNE plot (B). The pair of donut plots visually depict the distribution of different cell subpopulations identified through clustering analysis in the NC and IA groups (C). Scatter plots (D) depict the comparative proportions of each cell subpopulation between the two groups (each solid circle represents the proportion in a specific sample, the black dashed line indicates the mean of group and statistically significant differences are indicated by displayed *p* values). A ridge plot (E) illustrates the metabolic molecule expression in each cell subpopulation. Furthermore, a heatmap (F) unveils the patterns of functional molecule expression within each cell subpopulation.

To examine NK cell variations between NC and IA groups, isolated NK cells underwent Flowsom clustering. Seven subsets emerged, with NK03 and NK06 as CD56^bright^, while others were CD56^dim^ (Figures 7A and B).

NK02 accounted for the highest proportion in both NC and IA groups (NC: 40.92%; IA: 37.06%). NK06 constituted the least percentage in the NC group (3.41%), whereas NK07 held the lowest proportion in the IA group (4.04%) (Figure 7C). Comparing the proportions of various subpopulations between the two group, no significant statistical differences were observed (Figure 7D).

The metabolic profile of NK cells resembled that of NKT cells, with high expression of various metabolic molecules. Among them, NK06 stood out as a subset with elevated metabolism and function (Figures 7E and F). Compared with the NC group, NK06 and NK03 from the IA group exhibited increased expression of lipid metabolism molecules CD36, CPT1 and GLUT1 (Figure S7). Additionally, TCA‐related molecules IDH and SDH were also up‐regulated (IDH in NK06 without statistical significance between groups) (Figure S7). These cell subsets also demonstrated high proliferative capacity (Ki67, statistically different between groups) (Figure S7). The phenomenon of widespread elevation of the CD98 molecule in various NK cell subsets within the IA group was consistently observed. NK04 cells from the IA group exhibited high expression of CD36 (*p* = .00491 < .01) and mTOR (*p* = .0218 < .05), accompanied by enhanced proliferative capacity (KI67: *p* = .0109 < .05). NK05 cells were observed to have higher expression of CD36 (*p* = .0151 <.05) and SDH (*p* = .0238 < .05) in the IA group. Furthermore, NK01 cells from the IA group expressed more GLUT1 molecules (*p* = .0334 < .05) (Figure S7).

Functional analysis indicated that subsets derived from IA exhibited significantly higher expression of TNF‐α (statistical differences observed only in NK03, NK04 and NK06). Additionally, NK06 cells from the IA group were found to express more IL‐17 (*p* = .0166 < .05) (Figure S7).

### 3.7 Monocytes derived from IA displayed elevated expression of CD98, accompanied by a decrease in mTOR expression

**FIGURE 8:**
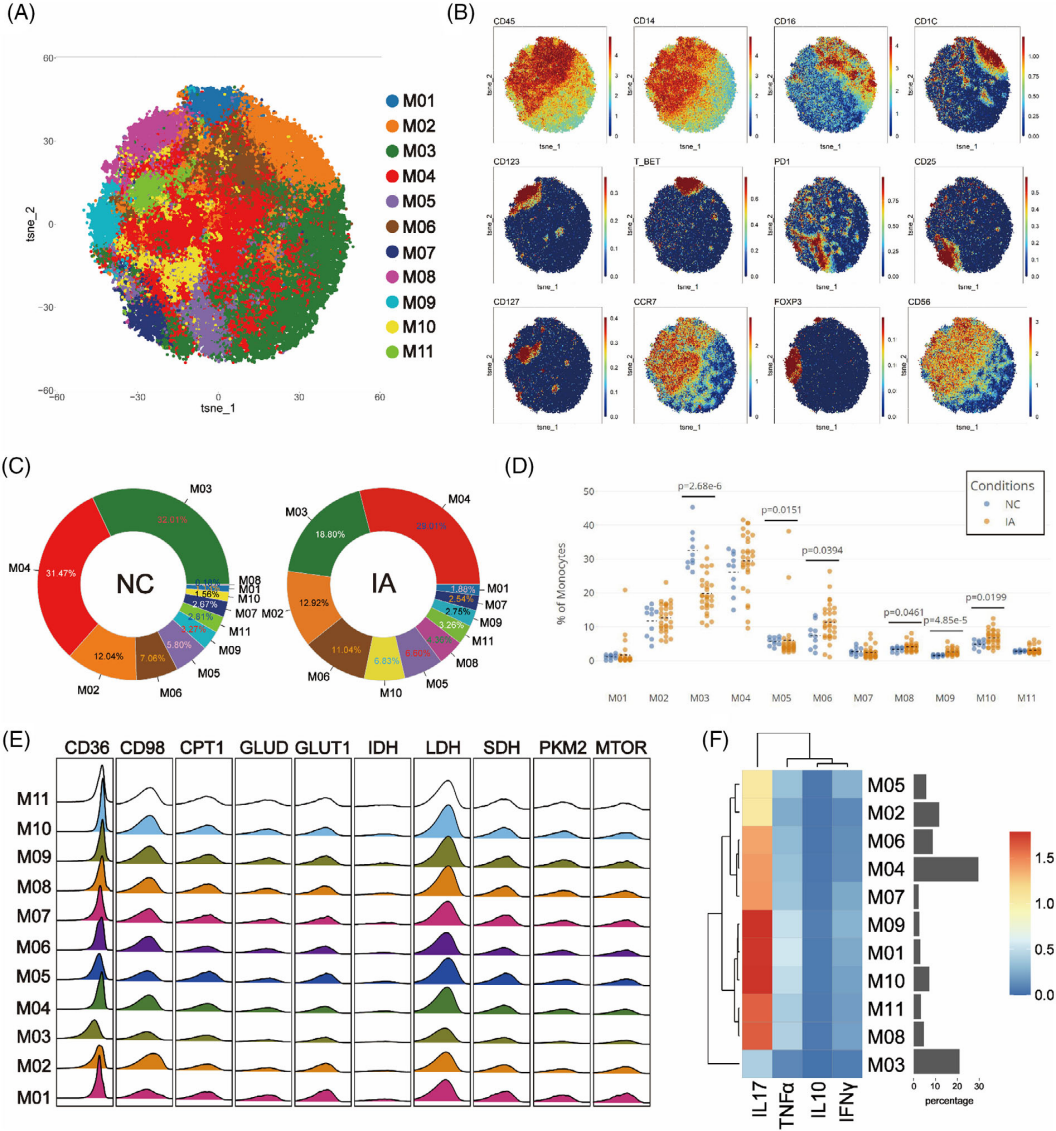
Comparative analysis of monocyte subsets. The T‐SNE plot displays the outcomes of the monocyte clustering analysis, with distinct subpopulations colour coded (A). Classic markers distinguishing these subpopulations are displayed in a spectral format on the T‐SNE plot (B). The two donut plots visually represent the proportions of various cell subpopulations identified in the NC and IA groups through clustering analysis (numerical values represent proportions, and colours align with the T‐SNE plot, arranged counterclockwise from high to low starting at 3 o'clock) (C). Scatter plots (D) illustrate the relative proportions of each subpopulation between the two groups (each solid circle indicated the proportion in a given sample, the black dashed line signifies the mean of group and statistically significant differences are indicated by displayed *p* values). A ridge plot (E) visually presents the expression of metabolic molecules within each subpopulation. Meanwhile, a heatmap (F) exhibits the expression pattern of functional molecules across each cell subpopulation.

In order to investigate differences between monocytes in the NC and IA groups, monocytes were isolated and subjected to Flowsom dimensionality reduction clustering analysis, revealing 11 distinct cell subsets (Figure 8A). Based on the expression of CD14 and CD16 molecules, M01 and M06 were categorised as CD14+CD16+ intermediate monocytes (iMo). M02 expressed CD1c, resembling a subset of myeloid dendritic cells (mDCs), while M08 expressed CD123, resembling a subset of plasmacytoid dendritic cells (pDCs). The remaining subsets were classified as CD14+CD16‐ classical monocytes (cMo) (Figure 8B).

M03 constituted the majority in the NC group (32.01%), whereas M04 was the dominant subset in the IA group (29.01%). M08 represented the smallest proportion in the NC group (0.18%), M01 was the cell subset with the least proportion of IA group (1.88%) (Figure 8C). In comparison with the NC group, M03, M04, M05, M07 and M09 exhibited increased proportions in monocytes from the IA group, while other subsets displayed decreased proportions (Figure 8C). Notably, significant differences were observed in the proportions of M03, M05, M06, M08, M09 and M10 between the two groups (Figure 8D).

The metabolic patterns of monocytes resembled those of T cells. Comparative analysis among subsets revealed that M02 displayed lower expression of metabolic and functional molecules, indicating a relatively lower metabolic state (Figures 8E and F). IA‐derived monocyte subsets still exhibited elevated CD98 expression alongside increased Ki67. mTOR was generally highly expressed in the NC group (Statistical differences were observed only in M05, M07 and M10.) (Figure S8). In addition, IDH (*p* = .00192 < .01), PD1 (*p* = .0334 < .05) and SDH (*p* = .0363 < .05) were highly expressed in the hypometabolism M02 subset in the IA group.

Functional analysis revealed elevated expression of TNF‐α and IL‐10 in monocyte subsets derived from IA, with the exception of M06 and M07, which did not reach statistical significance. Furthermore, it was observed that M04 (*p* = .0461 < .05) and M08 (*p* = .0199 < .05) from the IA group exhibited higher expression of IL‐17 (Figure S8).

### 3.8 The neutrophil spectrum in the IA group demonstrated a shift toward greater complexity

**FIGURE 9:**
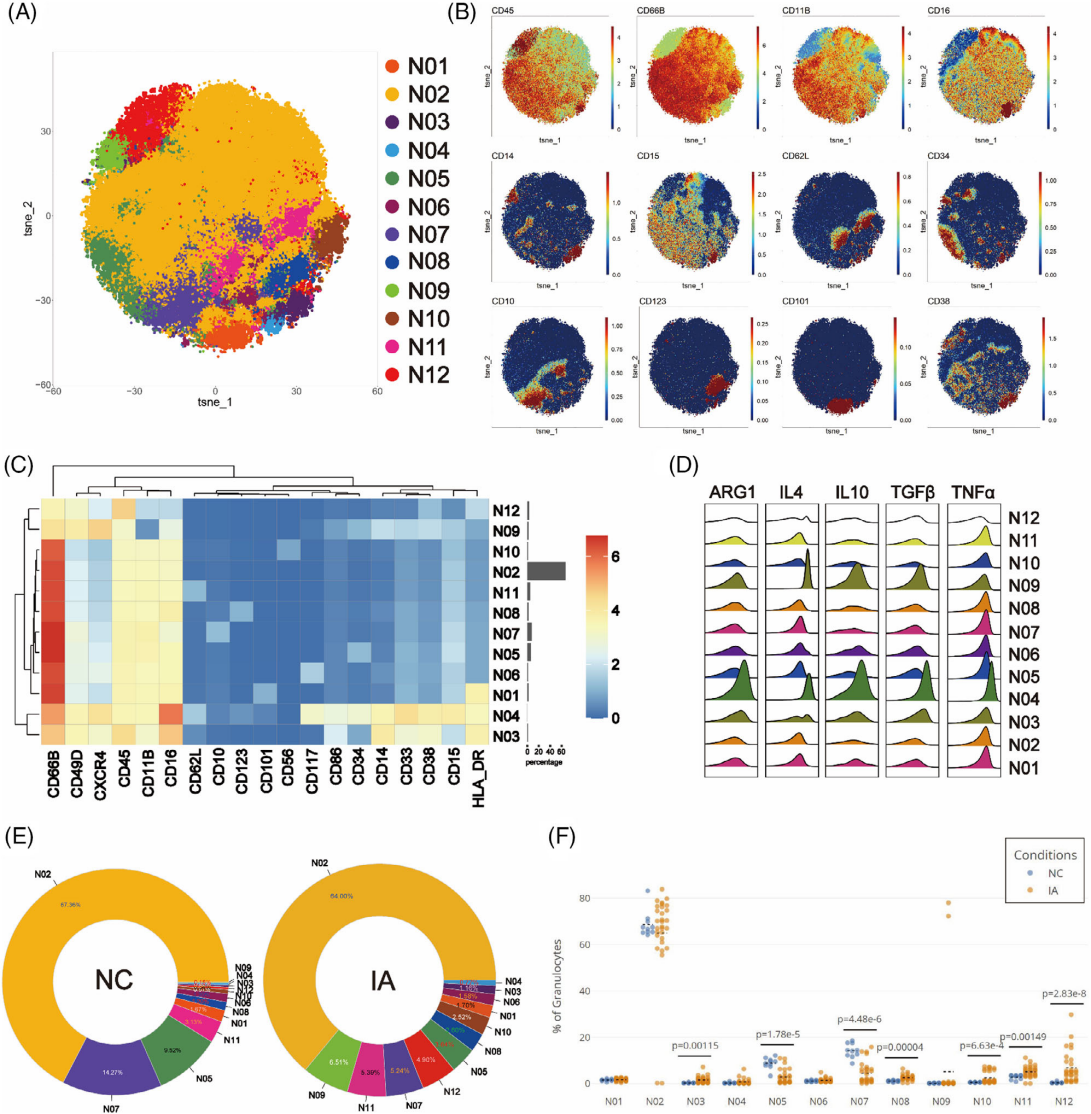
Comparative analysis of neutrophil subsets. The T‐SNE plot (A) illustrates the results of neutrophil clustering analysis, with distinct subpopulations colour coded. Classic markers distinguishing these subpopulations are presented in a spectral format on the T‐SNE plot (B). A heatmap (C) depicts the surface marker expression patterns of various cell subpopulations. A ridge plot (D) showcases the expression of functional molecules in each cell subpopulation. The two donut plots visually represent the proportions of various cell subpopulations identified in the NC and IA groups through clustering analysis (numerical values represent proportions, and colours align with the T‐SNE plot, arranged counterclockwise from high to low starting at 3 o'clock) (E). Scatter plots (F) illustrate the relative proportions of each subpopulation between the two groups (each solid circle indicated the proportion in a given sample, the black dashed line signifies the mean of group and statistically significant differences are indicated by displayed *p* values).

To investigate differences in PMNs between the NC and IA groups, isolated granulocytes were analysed using a dedicated antibody panel. The Flowsom clustering was similarly employed to explore subsets. A total of 12 clusters were identified (Figure 9A). Among these, N03, N04 and N09 expressed CD33 and CD14, resembling polymorphonuclear myeloid‐derived suppressor cells (PMN‐MDSCs). N03, which lacked the expression of the progenitor markers CD117 and CD34, belonged to a distinct functional subset. N01 and N12 both expressed the activation marker HLA‐DR, indicating specialised functional states. CD34, CD117 and CD123 were respectively expressed in N05, N06 and N08, suggesting their affiliation with immature subsets, while mature markers CD10 and CD101 were present on the surfaces of N07 and N01, designating these two clusters as mature subsets. N02 displayed typical neutrophil surface markers (CD66b+CD11b+CD15+CD14−CD16+). Furthermore, N10 expressed CD56, indicating distinct cellular subsets (Figures 9B and C). N11 not only expressed surface markers characteristic of neutrophils but also exhibited CD62L expression. Comparative analysis of different clusters revealed heightened expression of all functional molecules in N09 except for TNF‐α, while N04 displayed elevated expression of all functional molecules (Figure 9D).

Regardless of NC or IA groups, N02 constituted the largest proportion among neutrophil subsets (NC: 67.36%; IA: 64.00%). N09 represented the least abundant subset in NC (0.15%), while N04 held the lowest proportion in IA (0.77%) (Figure 9E). In comparison to the NC group, the proportions of most subsets from IA group increased, except for N02, N05 and N07 which decreased (Figure 9E). When comparing the proportions of various subsets between the two groups, significant differences were observed among N03, N05, N07, N08, N10, N11 and N12 (Figure 9F).

Functional analysis revealed that various subsets in the NC group exhibited higher expression of TNF‐α, IL‐4 and TGF‐β compared to the IA group (with some not reaching statistical significance but displaying trends) (Figure S9). Except for N05 and N06, ARG1 was also generally more expressed in the NC group (with significant differences observed in N09, N10 and N12). Notably, N04 (*p* = .0238 < .05), N09 (*p* = .0196 < .05) and N12 (*p* < .001) also displayed elevated levels of IL‐10. Interestingly, N07 (*p* < .001) and N10 (*p* = .0363 < .05) from the IA group exhibited higher IL‐10 expression compared with the NC group (Figure S9).

### 3.9 Distinct immune cell interactions between NC and IA group

To comprehensively analyse whether there are differences in the correlations between immune cell subsets in the NC and IA groups, we conducted a correlation analysis of the proportions of major immune cell subsets (major cell types, high and low metabolism subsets and clusters with significant metabolic disparities) identified by CyTOF.

**FIGURE 10:**
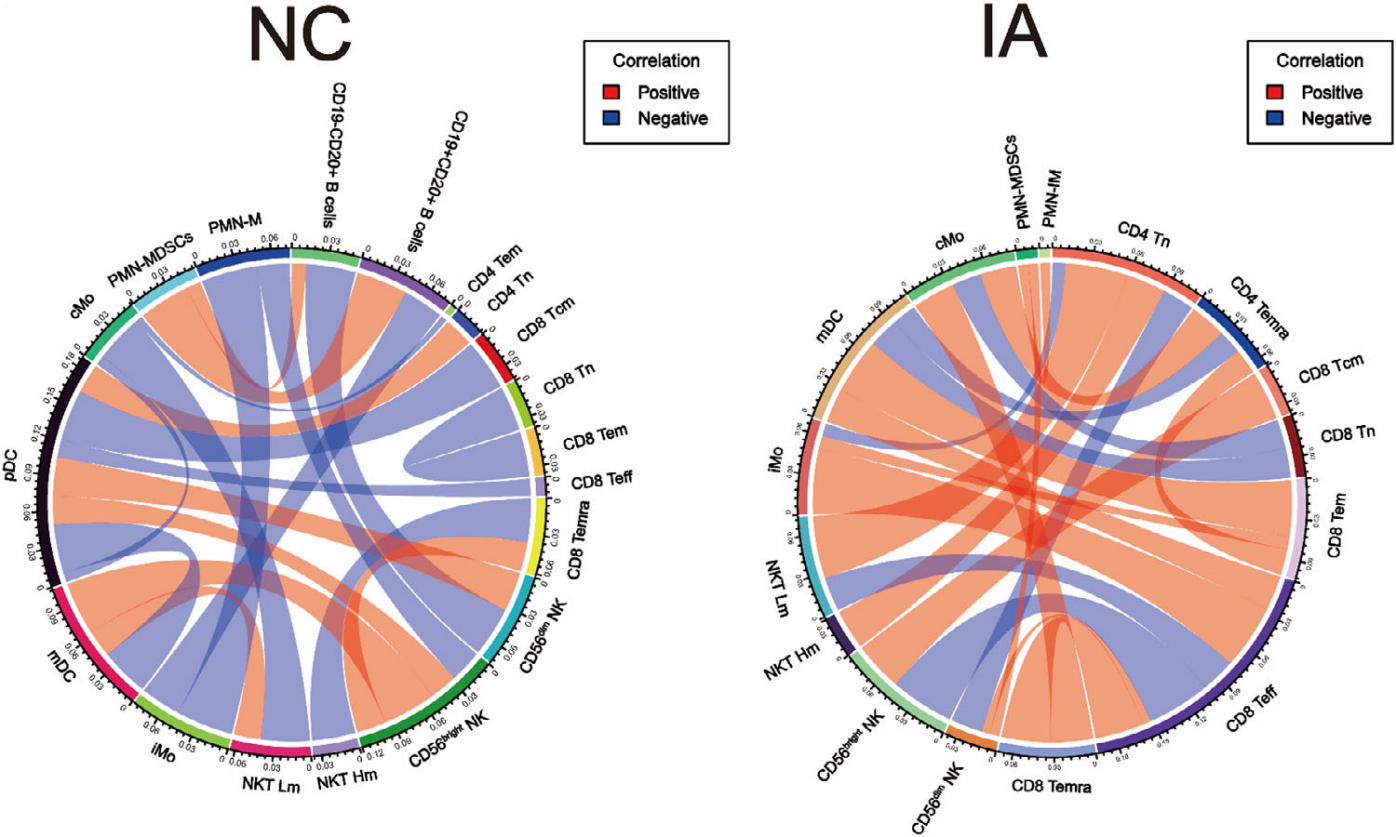
Peripheral blood immune cell correlation analysis. The Circos plot illustrates the statistically significant correlations between major cell subsets in peripheral blood of NC and IA group (red lines represent positive correlations, while blue lines represent negative correlations).

Overall, correlations within the IA group appeared to be more complex and diverse. The cellular associations within the IA group predominantly showed positive correlations, whereas the NC group exhibited more negative correlations. Correlations were primarily associated with pDCs in the NC group. Specifically, pDCs exhibited positive correlations with CD4 Tn and NK cells (CD56^bright^ NK and CD56^dim^ NK), while displaying negative correlations with CD8 Tcm, CD8 Teff, mDCs and cMo. Additionally, mDCs showed positive correlations with CD56^bright^ NK and NKT Lm (low‐metabolism NKT cells, excluding NKT03). iMo exhibited negative correlations with CD19+CD20+ B cells and PMN‐M (mature PMNs, subsets except for MDSCs and IM). PMN‐MDSCs displayed positive correlations with B cells (CD19+CD20+ B cells and CD19−CD20+ B cells). CD8 Temra showed negative correlations with NKT Hm (high‐metabolism NKT cells, NKT03) and positive correlations with CD56^bright^ NK. cMo exhibited correlations with NKT Hm and CD4 Tem, CD56^bright^ NK with PMN‐M, CD56dim NK with CD19‐CD20+ B cells, and both CD8 Tem and CD8 Tn displayed negative correlations (Figure 10, left).

However, correlations within the IA group primarily revolved around CD8 Teff, mDC and CD4 Tn. Specifically, CD8 Teff exhibited positive correlations with CD8 Temra, CD56^dim^ NK, iMo and mDC, while displaying negative correlations with CD56^bright^ NK and NKT Lm. mDC showed positive correlations with CD8 Tem, but negative correlations with CD8 Tn and CD4 Temra. CD4 Tn exhibited positive correlations with NKT Hm and NKT Lm, while displaying negative correlations with CD56^dim^ NK and iMo. Additionally, we observed a positive correlation between cMo and CD4 Temra, and a negative correlation between cMo and CD8 Tn. CD8 Tem showed positive correlations with CD4 Temra and iMo. Furthermore, PMN‐IM exhibited positive correlations with PMN‐MDSCs and CD56^dim^ NK (Figure 10, right).

### 3.10 Establishment and evaluation of IA occurrence and growth models

Based on the analysis of CyTOF results, we have identified a distinctive immune landscape in the peripheral blood of IA patients. The relative proportions of various immune cell subsets in peripheral blood appear to have predictive value for the occurrence and progression of IAs. Consequently, we integrated these features with clinical information to separately establish prediction models for the occurrence and growth of IAs. These models aim to assist in clinical diagnosis and disease progression monitoring.

**FIGURE 11:**
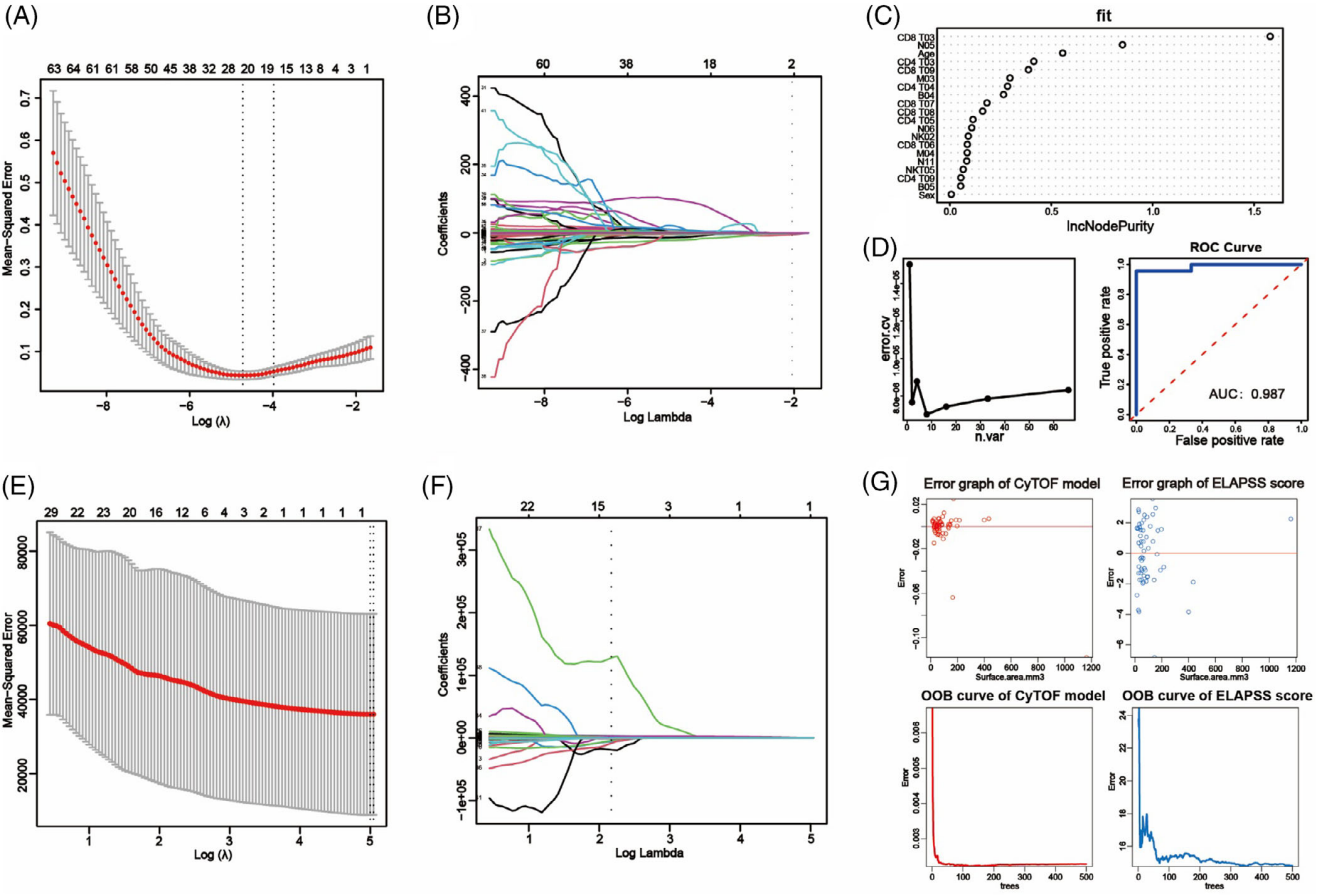
Occurrence and growth models of IA. Occurrence and growth models. The LASSO parameter plot and LASSO coefficient plot illustrate the variable selection process for the intracranial aneurysm occurrence model, with 20 variables included at the minimum *λ* value (A and B). The scatter plot presents the contribution of variables selected by LASSO regression to the random forest tree model in descending order (C). The line graph depicts the optimisation of the model through fivefold cross‐validation recursive methods (D left, the minimum error rate achieved when the model incorporates eight variables). The ROC curve demonstrates the predictive performance of the optimised aneurysm occurrence model in the test set (D right). In the same way, the LASSO parameter plot and LASSO coefficient plot illustrate the variable selection process for the growth model (E and F). Prediction error maps and OBB error maps illustrate the differences in predictive performance between the growth model and ELAPSS score (G).

For the IA occurrence model, we initially employed LASSO regression to screen factors associated with aneurysm from clinical features and immune characteristics identified through CyTOF. The analysis revealed the significance of 20 factors, including B04, B05, CD4 T03, CD4 T04, CD4 T05, CD4 T09, CD8 T03, CD8 T06, CD8 T07, CD8 T08, CD8 T09, NK02, NKT05, M03, M04, N05, N06, N11, Sex and Age (Figures 11A and B). Seventy percent of the samples were randomly selected as the training set, with the remaining 30% designated as the test set. In the training set, a fivefold cross‐validation approach was employed to train the model. Through a recursive method, the model demonstrated the lowest error rate (error = 7.019492e−06) when incorporating eight variables (Figure 11D, left). Based on the weighted ranking of model variables, the top eight variables were selected for optimisation (including CD8 T03, N05, Age, CD4 T03, CD8 T09, M03, CD4 T04 and B04) (Figure 11C). Subsequently, the predictive performance of the model was assessed in the test set, revealing its ability to effectively identify IA patients (AUC = 0.987) (Figure 11D, right).

To investigate which subsets were associated with the development of aneurysms, Vascular reconstruction technology was utilised to measure the surface area of aneurysms in 58 patients with complete preoperative imaging information (Figure S2). Subsequently, employing a similar methodology, we initially applied LASSO regression to identify factors associated with the surface area of aneurysms. Ultimately, N03 was identified as closely related to aneurysm surface area (Figures 11E and F). Subsequently, a random forest tree model was constructed to assess the relationship between aneurysm surface area and N03. We also used ELAPSS scores to evaluate aneurysm growth risk. By comparing the predictive errors of both models, it was observed that the CyTOF model exhibited a narrower error distribution compared to the ELAPSS score (Figure 11G, upper). This suggests that the CyTOF model possesses higher predictive accuracy. Furthermore, as the number of trees increased, the OBB error of the CyTOF model consistently converged, further indicating the superior predictive performance of CyTOF over the ELAPSS score (Figure 11G, lower).

## 4 DISCUSSION

IAs are found in approximately 1–2% of the population and commonly arise at the intersections of intracranial arteries.^3^, ^27^, ^28^ SAH induced by IAs can lead to a poor outcome in up to 35% of patients.^29^ Comprehending the mechanisms underlying the development of IAs contributes to preventing their occurrence and progression. In this study, CyTOF technology was employed to comprehensively analyse the PBMCs and PMNs between healthy individuals and patients with IA. Furthermore, correlation analysis was used to explore associations among different subsets and prediction models were established to assist clinical diagnosis and progression monitoring of IAs. These findings offered valuable insights into the potential peripheral immune mechanisms underlying the occurrence and progression of IA, providing important perspectives for the development of targeted preventive measures.

Inflammatory processes play a significant role in the development of IA.^30^, ^31^ Several research exploring the association between chronic inflammation and IAs suggested that persistent disruption of the peripheral immune environment may induce the formation of IAs through inflammatory cascades.^32^, ^33^, ^34^, ^35^ In the present, it was observed that PBMCs, as a crucial component of the peripheral immune environment, exhibited metabolic dysregulation associated with numerous diseases. In our study, metabolic profiling of PBMCs revealed distinct metabolic characteristics among different subsets. Monocytes and T cells exhibited similar metabolic patterns, while B cells exhibited high expression of mTOR. Both NK cells and NKT cells were characterised by multiple metabolic energy sources. In the same PBMCs population, there coexist multiple subsets with distinct metabolic states. The heightened metabolic state was correlated with enhanced functional attributes.^36^, ^37^ Overall, high‐metabolic subsets were predominantly concentrated in the peripheral blood of IA patients, while low‐metabolic immune cells were primarily found in the peripheral blood of the NC group. Relative to the NC group, CD98 was found to be widely expressed in various subpopulations of PBMCs from IA patients. As the l‐amino acid transporter, CD98 regulated cellular amino acid uptake and protein synthesis.^38^ Furthermore, research has indicated the pivotal role of CD98 in sustaining the activated state of immune cells.^39^ Its widespread higher expression in immune cell subsets derived from IA suggested that immune cells in the peripheral blood of IA patients exhibited elevated synthetic metabolism and proliferative characteristics. This corresponded to the fact that Ki67 was generally overexpressed in IA. Comparative analysis also revealed that, except for NK and NKT cells, CD36 was generally under‐expressed in PBMCs from the IA group. CD36 as both a signal transducer and fatty acid transporter also control immune cell fate.^40^, ^41^ Studies indicated that CD36 was indispensable for maintaining the normal proliferative activity of stem cells, enabling monocytes to perform their functions and ensuring the survival of Treg cells.^42^, ^43^, ^44^ The high expression of CD36 in the NC group may be associated with the stability of the peripheral immune microenvironment. Meanwhile, the widespread overexpression of CD36 in NK and NKT cell subpopulations in the IA group could be related to the higher metabolic demands of these cells in this environment. This provided these cells with more substrates for mitochondrial oxidative phosphorylation. The differential expression of these metabolism‐related molecules in PBMCs reflects the distinct metabolic transition patterns of various immune cells, potentially influencing the systemic immune environment and contributing to the development of IAs. In cell cross‐linking analysis, we observed widespread positive correlations among immune cells in IA peripheral blood, contrasting with the prevalent negative correlations in the peripheral blood of the NC group. Notably, mDCs in the peripheral blood of IA patients may play a crucial role in inflammation regulation. Further metabolic analysis revealed up‐regulation of SDH and IDH in mDC cells in the peripheral blood of IA patients, potentially driving their enhanced pro‐inflammatory activity in the peripheral environment of IAs. Adropin, an anti‐inflammatory secreted protein, influences cell metabolism by interfering with the activity of key enzymes in glycolysis and fatty acid oxidation.^45^, ^46^ Additionally, targeting specific microRNAs can affect the expression of intracellular metabolic enzymes.^47^, ^48^ These advancements enable molecular‐level modulation of the metabolic activity of peripheral immune cells, potentially reversing the immune cascade reactions in peripheral blood. Thus, the development of drugs promoting the metabolic transition of T cells, B cells and monocytes in peripheral blood may prevent the occurrence and progression of aneurysms.

Recent studies have unveiled substantial phenotypic and functional heterogeneity among neutrophils, traditionally considered a homogeneous population.^49^, ^50^ Our study has also illuminated the presence of multiple neutrophil subsets within the peripheral blood of patients with IA. These cells can be roughly classified into PMN‐MDSCs, mature neutrophils and immature neutrophils, with the highest abundance observed in mature neutrophils (N02). In contrast to the NC group, various PMNs subsets from the IA group generally exhibited lower levels of anti‐inflammatory functional molecules. Statistical modelling revealed that immature neutrophils expressing CD34 (N05) were a protective factor against the occurrence of IAs. Studies have shown that CD34+ cells possess the capability to migrate to damaged tissues and promote repair and regeneration.^51^ This suggests that these cells may serve as a potential protective factor against the development of aneurysms. Furthermore, we also observed a close association between N03 (identified as a subset of PMN‐MDSCs) and the surface area of the aneurysm. MDSCs are generally considered a heterogeneous population of cells associated with immune suppression and tolerance.^52^, ^53^ The expansion of MDSCs is often regarded as a protective mechanism that can, to some extent, dampen immune responses. However, extensive research suggested that as diseases progress, the production of MDSCs actually increases.^54^, ^55^, ^56^ This might be a response phenomenon to the severity of the disease. In our study, the proportion of N03 cells in peripheral blood proved to be a superior indicator for predicting aneurysm growth compared with the ELAPSS aneurysm growth score.

## 5 CONCLUSION

In our study, we presented a detailed single‐cell metabolic atlas of PBMCs and single‐cell profiles of PMNs in IA patients. Through comparison with healthy individuals, we identified metabolic alterations in certain PBMC subsets and changes in the distribution of neutrophil subgroups in IA patients. These alterations may potentially impact the occurrence and progression of IAs and provided valuable insights for the development of targeted therapeutic interventions.

## AUTHOR CONTRIBUTIONS

X. Y. collected blood samples and performed single‐cell suspension extraction. X. Y., C. L. and L. M. performed the extraction of PBMCs and PMNs. P. C. gave some advice. Z. Z. and S. M. helped organise some of the data. Y. Z., R. W., Q. Z. and X. Y. provided the guidance for this experiment. W. J. W., H. L. and J. Z. supervised this research.

## CONFLICT OF INTEREST STATEMENT

The authors declare no conflict of interest.

## ETHICS STATEMENT

This study received approval from the Institutional Review Board (IRB) and Ethics Committee of Beijing Tiantan Hospital (Beijing, China) (KY2017‐035‐02). Written informed consent was obtained from all participating patients and healthy controls.

## Supporting information

Supporting Information

Supporting Information

## Data Availability

The data that support the findings of this study are available from the corresponding author upon reasonable request.
