# Supplementary material for: Metabolic alterations of peripheral blood immune cells and heterogeneity of neutrophil in intracranial aneurysms patients
Source: Clin Transl Med. 2024 Feb 5;14(2):e1572. doi: 10.1002/ctm2.1572 (PMC10840020; doi:10.1002/ctm2.1572)
Supplement: Supplementary file 1 — Supporting Information [file CTM2-14-e1572-s001.docx]

| \| **Supplementary Table 1. Clinical characteristics of 72 IA patients**   \| **Parameters** \| **Patients (n=72)** \| \| --- \| --- \| \| **Basic characteristics** \| \| \| Gender (male/female) \| 33/39 \| \| Age (year, Median/IQR) \| 55.5/16.5 \| \| **History** \| \| \| Drinking, n(%) \| 14(19.44%) \| \| Smoking, n(%) \| 14(19.44%) \| \| Hypertension, n(%) \| 43(59.72%) \| \| Dyslipidemia, n(%) \| 6(1.39%) \| \| Drinking, n(%) \| 14(19.44%) \| \| \| --- \| --- \| --- \| --- \| --- \| --- \| --- \| --- \| --- \| --- \| --- \| --- \| --- \| --- \| --- \| --- \| --- \| --- \| --- \| --- \| --- \|   **Supplementary Table 2. Clinical characteristics of 30 IA patients and 10 healthy controls** | | | |
| --- | --- | --- | --- | --- | --- | --- | --- | --- | --- | --- | --- | --- | --- | --- | --- | --- | --- | --- | --- | --- | --- | --- | --- | --- |
| **Parameters** | **Controls (n=10)** | **Patients (n=30)** | **P-value** |
| **Basic characteristics** |  |  |  |
| Gender (male/female) | 4/6 | 13/17 | 0.854 |
| Age (year, Median/IQR) | 46/8.5 | 49.5/8.5 | 0.201 |
| **History** | | | |
| Drinking, n(%) | 2(20.0%) | 5(16.7%) | 0.810 |
| Smoking, n(%) | 1(10.0%) | 6(20.0%) | 0.471 |
| Hypertension, n(%) | 3(40.0%) | 18(60.0%) | 0.100 |
| Dyslipidemia, n(%) | 0(0%) | 3(10.0%) | 0.299 |
| Diabetes, n(%) | 0(0%) | 7(23.3%) | 0.093 |

* p < 0.05.

**Supplementary Table 3. Mass cytometry antibodies panel of PBMCs.**

| **Antigen** | **Symbol and Mass** | **Antibody clone** | **Source** |
| --- | --- | --- | --- |
| CD45 | 89 | HI30 | Fluidigm |
| CD3 | 111 | Hu113 | R＆D |
| CD4 | 116 | 34930 | R＆D |
| PKM2 | 141 | EPR10138(B) | abcam |
| CD19 | 142 | HIB19 | Fluidigm |
| CCR6 | 143 | 53103 | R＆D |
| GLUT1 | 145 | SP168 | abcam |
| CD45RA | 146 | HI100 | Fluidigm |
| CD20 | 147 | 2H7 | Fluidigm |
| mTOR | 148 | EPR427(N) | abcam |
| CD25 | 149 | 2A3 | Fluidigm |
| SDH | 150 | EPR9043(B) | abcam |
| PD-1 | 151 | 913429 | R＆D |
| CD123 | 152 | 32703 | R＆D |
| CPT1 | 153 | EPR21843-71-1C | abcam |
| LDH | 154 | EP1565Y | abcam |
| GLUD | 156 | EPR11369(B) | abcam |
| IDH | 158 | EPR21002 | abcam |
| CCR7 | 159 | G043H7 | Fluidigm |
| TNF-a | 160 | EPR20972 | abcam |
| T-bet | 161 | EPR27094-16 | abcam |
| CD1c | 162 | L161 | Biolegned |
| CD66b | 163 | 913542 | R＆D |
| CD98 | 164 | MEM-108 | Biolegned |
| CD127 | 165 | A019D5 | Fluidigm |
| IL-10 | 166 | EPR1114 | abcam |
| GATA-3 | 167 | EPR16651 | abcam |
| FoxP3 | 169 | EPR22102-37 | abcam |
| IL-17 | 170 | QA18A46 | Biolegned |
| CD45RO | 171 | UCHL1 | Biolegned |
| CD36 | 172 | 255606 | R＆D |
| CD56 | 173 | 301021 | R＆D |
| CD14 | 174 | 134620 | R＆D |
| CD8 | 175 | 37006 | R＆D |
| IFN-r | 176 | EPR23991-53 | abcam |
| CD16 | 209 | 3G8 | Fluidigm |

**Supplementary Table 4. Mass cytometry antibodies panel of PMNs.**

| **Antigen** | **Symbol and Mass** | **Antibody clone** | **Source** |
| --- | --- | --- | --- |
| CD45 | 89 | HI30 | Fluidigm |
| CD49d | 141 | 9F10 | Fluidigm |
| IL-4 | 142 | MP4-25D2 | Fluidigm |
| CD11b | 144 | ICRF44 | Fluidigm |
| CD86 | 147 | 37301 | R＆D |
| CD15 | 148 | ICRF29-2 | R＆D |
| CD34 | 149 | 581 | Fluidigm |
| CD10 | 150 | 212504 | R＆D |
| Arg-1 | 151 | EPR10411 | abcam |
| CD123 | 152 | 32703 | R＆D |
| CD62L | 153 | DREG-56 | Fluidigm |
| CD101 | 155 | BB27 | Biolegned |
| HLADR | 156 | L203 | R＆D |
| CD33 | 158 | WM53 | Fluidigm |
| CD56 | 159 | 301021 | R＆D |
| TNF-a | 160 | EPR20972 | abcam |
| CD66b | 163 | 913542 | R＆D |
| IL10 | 166 | EPR1114 | abcam |
| CXCR4 | 167 | 44716 | R＆D |
| CD14 | 169 | 134620 | R＆D |
| CD117 | 170 | 47233 | R＆D |
| CD38 | 174 | S17015F | Biolegned |
| TGFb | 176 | EPR12079(B) | abcam |
| CD16 | 209 | 3G8 | Fluidigm |
