# Supplementary material for: Metabolic alterations of peripheral blood immune cells and heterogeneity of neutrophil in intracranial aneurysms patients
Source: Clin Transl Med. 2024 Feb 5;14(2):e1572. doi: 10.1002/ctm2.1572 (PMC10840020; doi:10.1002/ctm2.1572)
Supplement: Supplementary file 2 — Supporting Information [file CTM2-14-e1572-s002.docx]

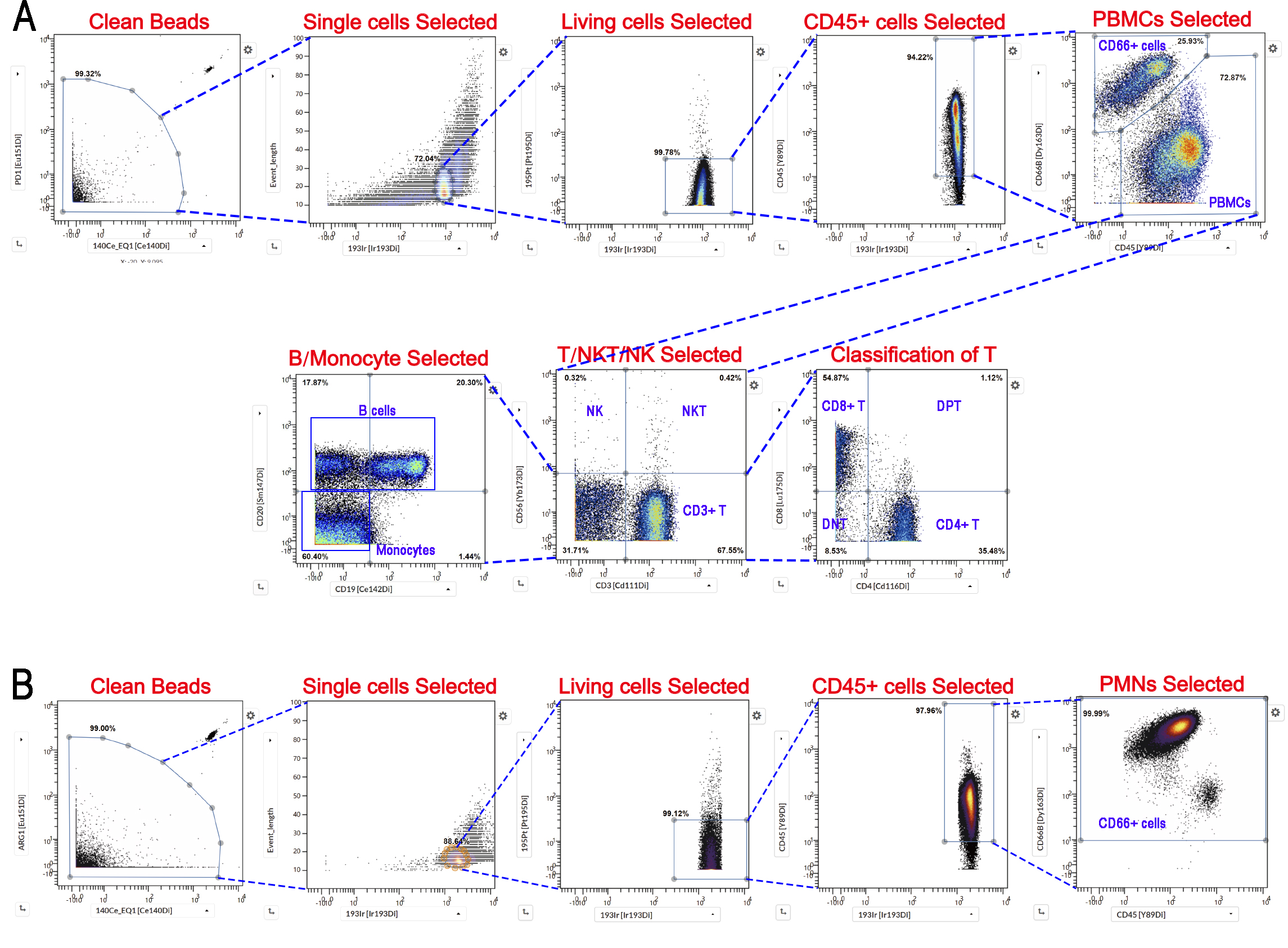


**Supplementary Figure 1:** CyTOF data preprocessing and cell subset isolation strategies for PBMCs before Flowsom clustering analysis (A). CyTOF data preprocessing and isolation for PMNs (B).


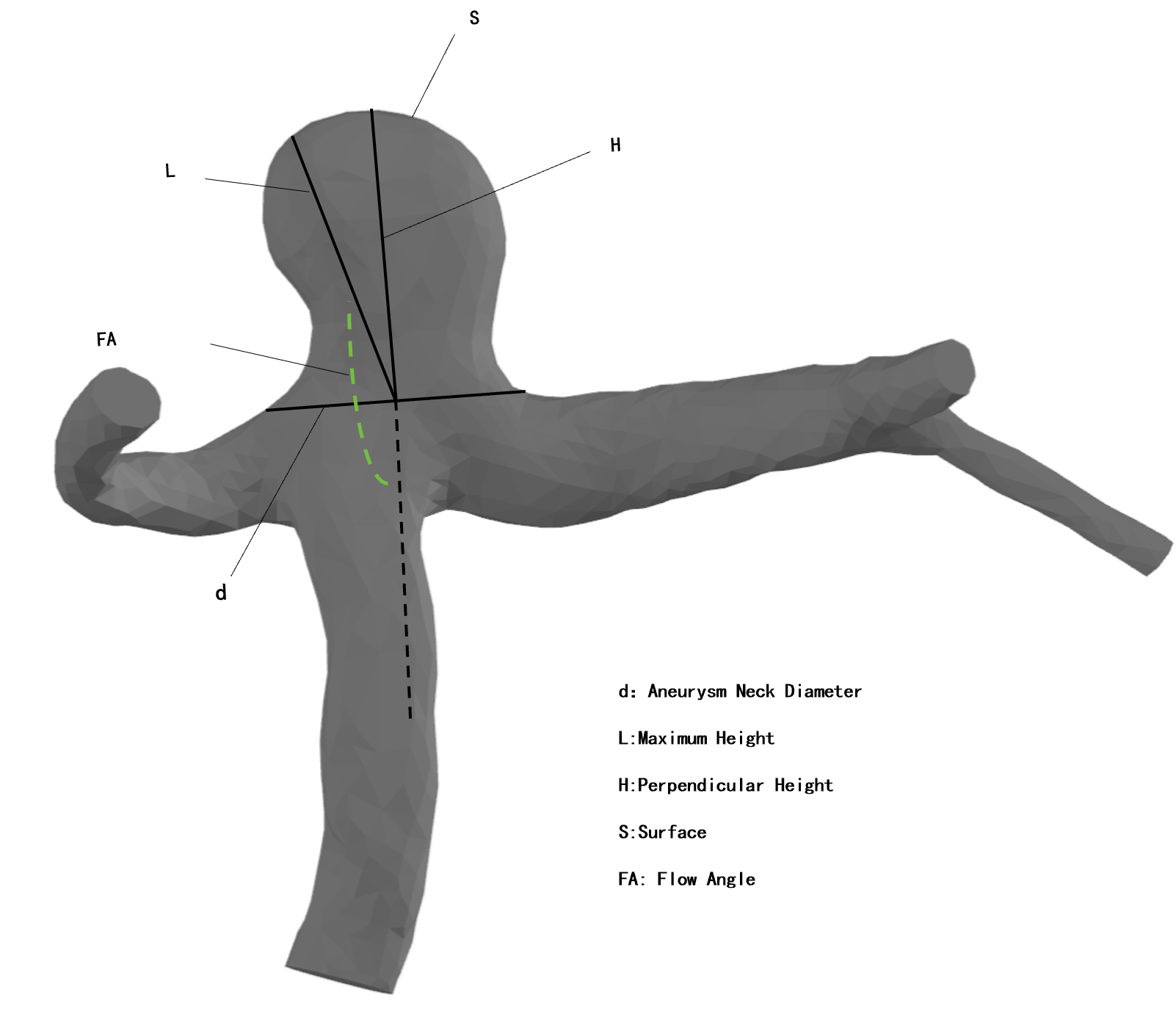


**Supplementary Figure 2:** Diagram illustrating aneurysm morphological parameters. (**H**) represents the maximum vertical distance from the dome to the neck plane. (**L**) indicates the maximum distance from the dome to the neck plane. (**d**) represents the average diameter at the neck. (**s**) denotes the surface area of the aneurysm sac. (**FA**) stands for flow angle. The flow angle is defined as the angle between the inlet vessel centerline and the maximum length.


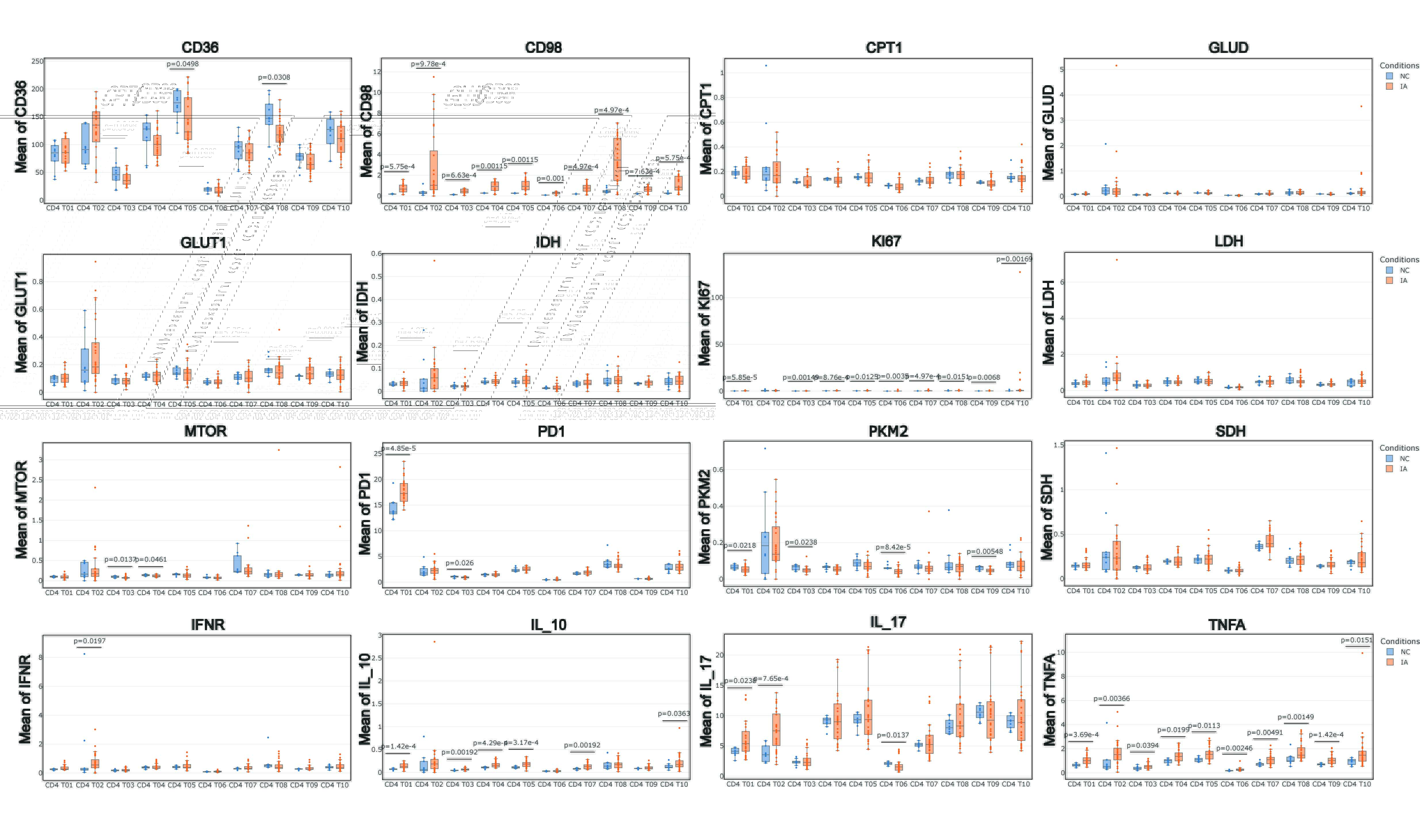


**Supplementary Figure 3:** Comprehensive comparison of functional and metabolic molecules among various CD4+ T cell subsets between NC and IA groups.


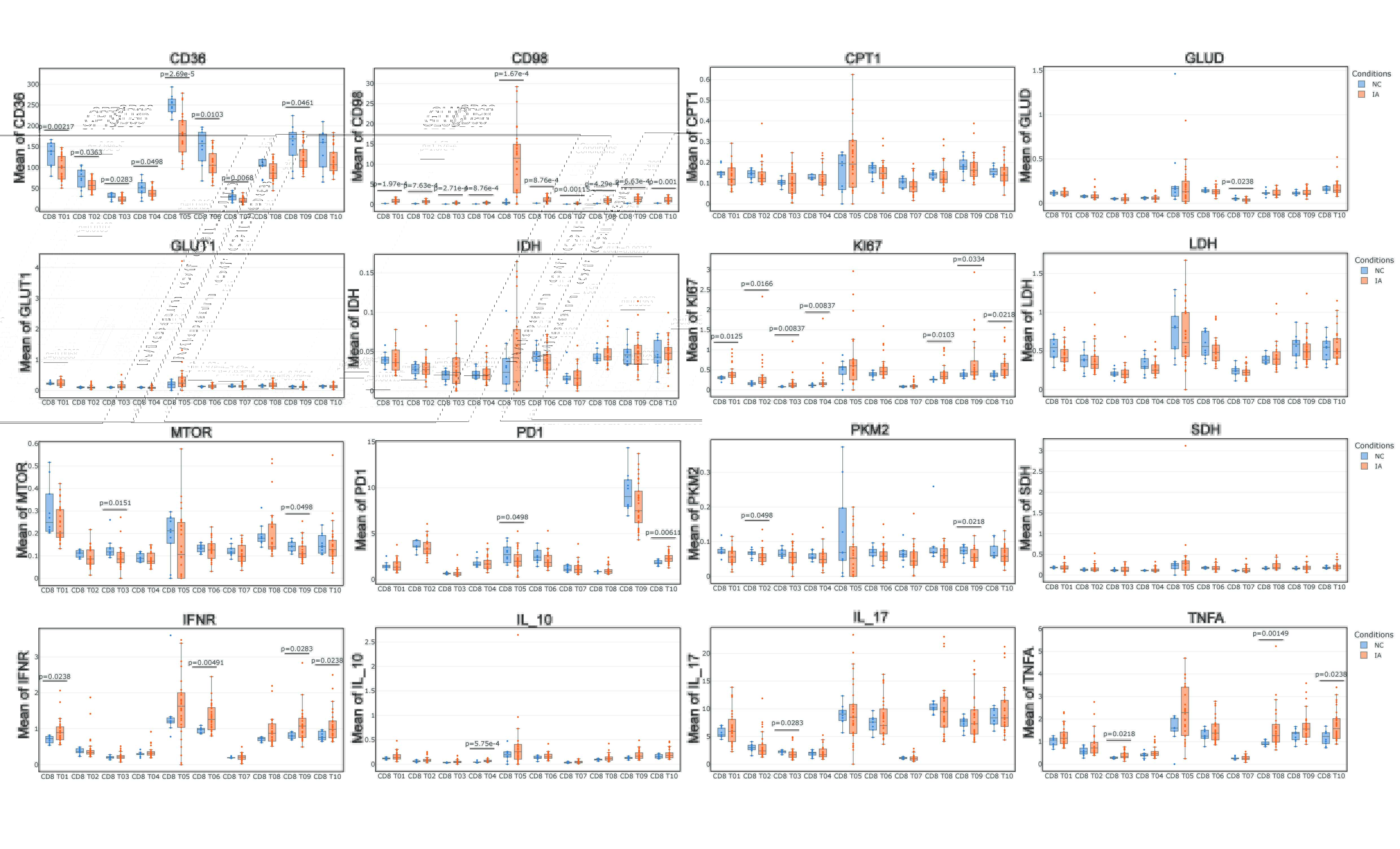


**Supplementary Figure 4:** Comprehensive comparison of functional and metabolic molecules among various CD8+ T cell subsets between NC and IA groups.


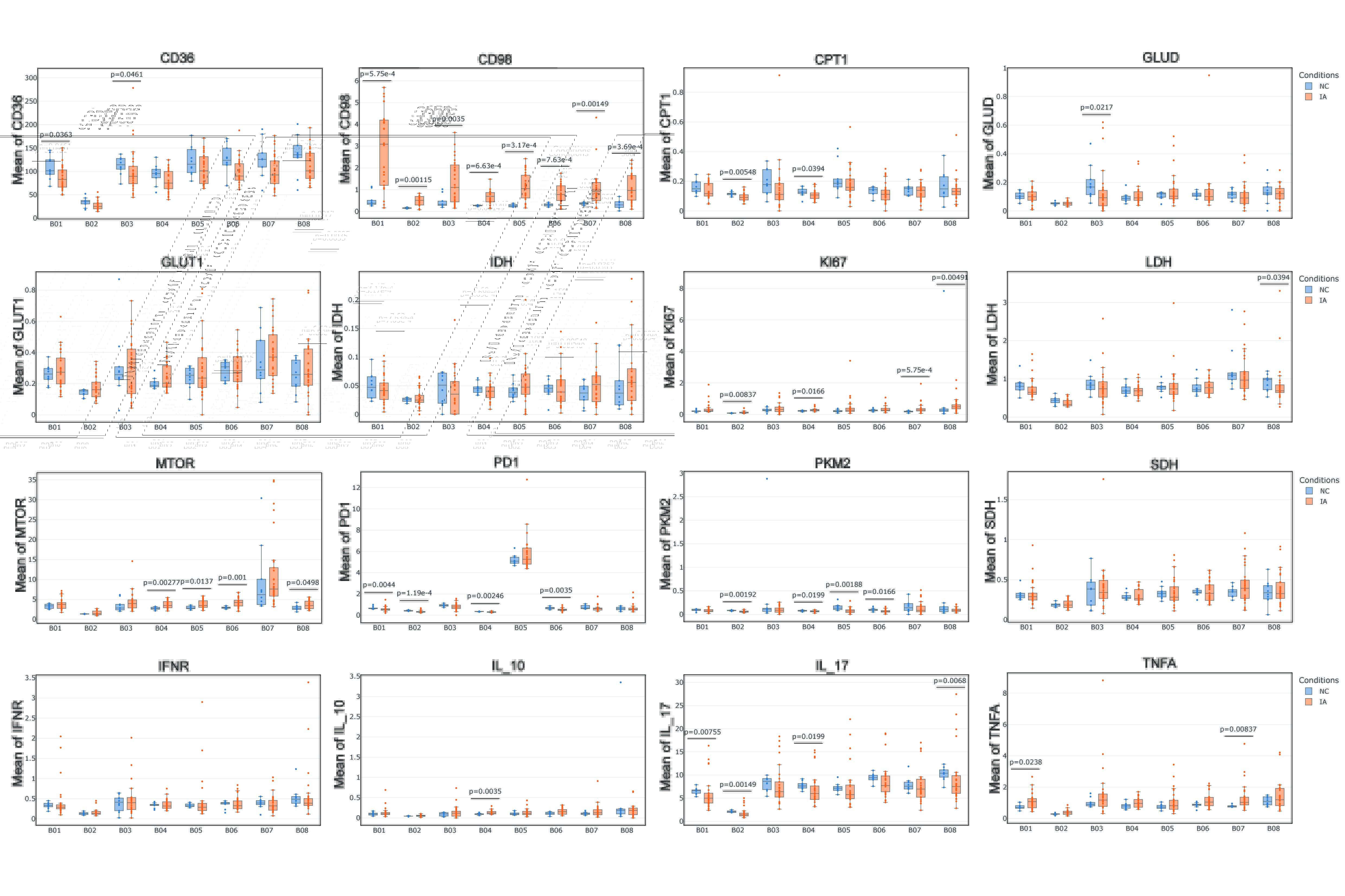


**Supplementary Figure 5:** Comprehensive comparison of functional and metabolic molecules among various B cell subsets between NC and IA groups.


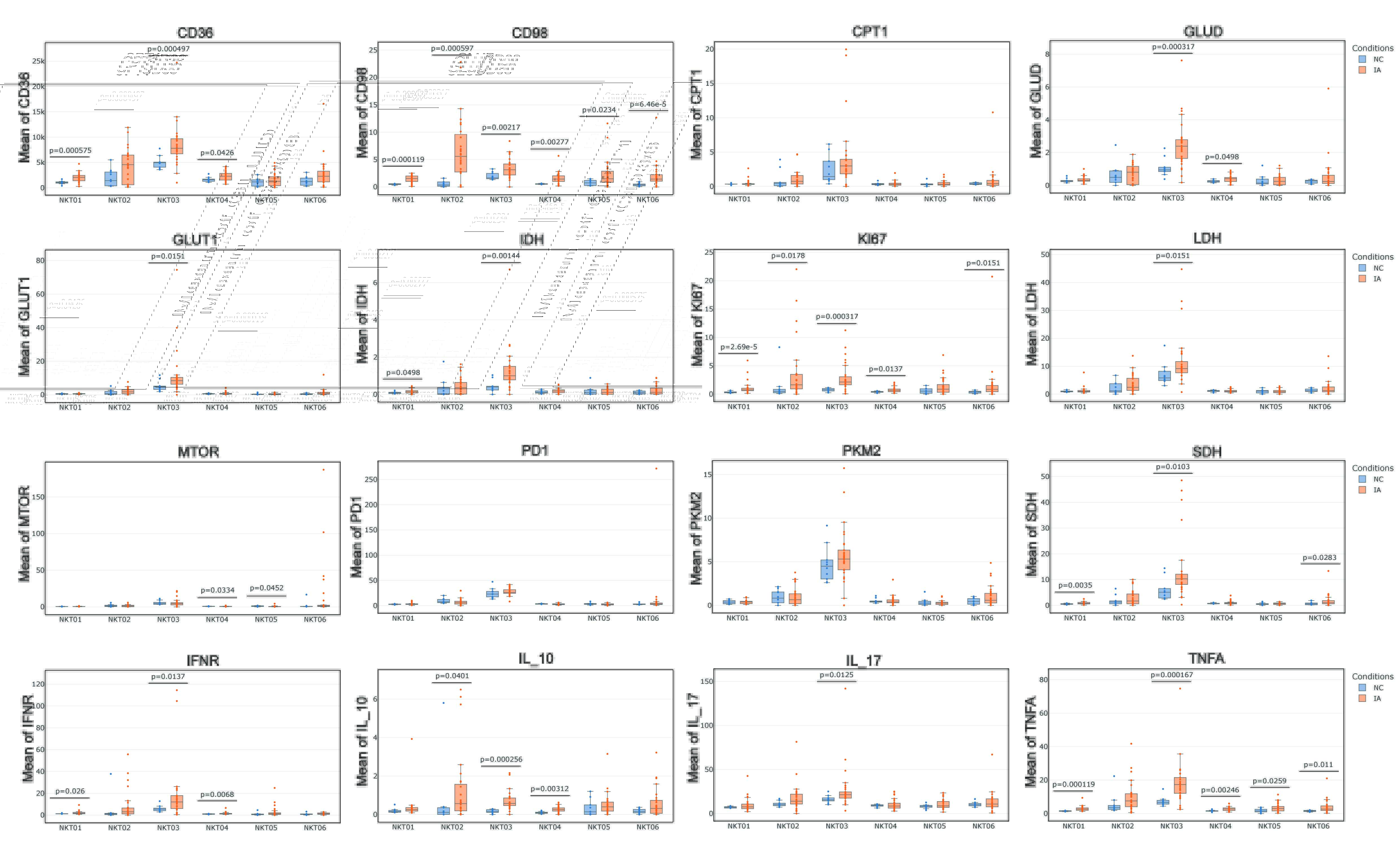


**Supplementary Figure 6:** Comprehensive comparison of functional and metabolic molecules among various NKT subsets between NC and IA groups.


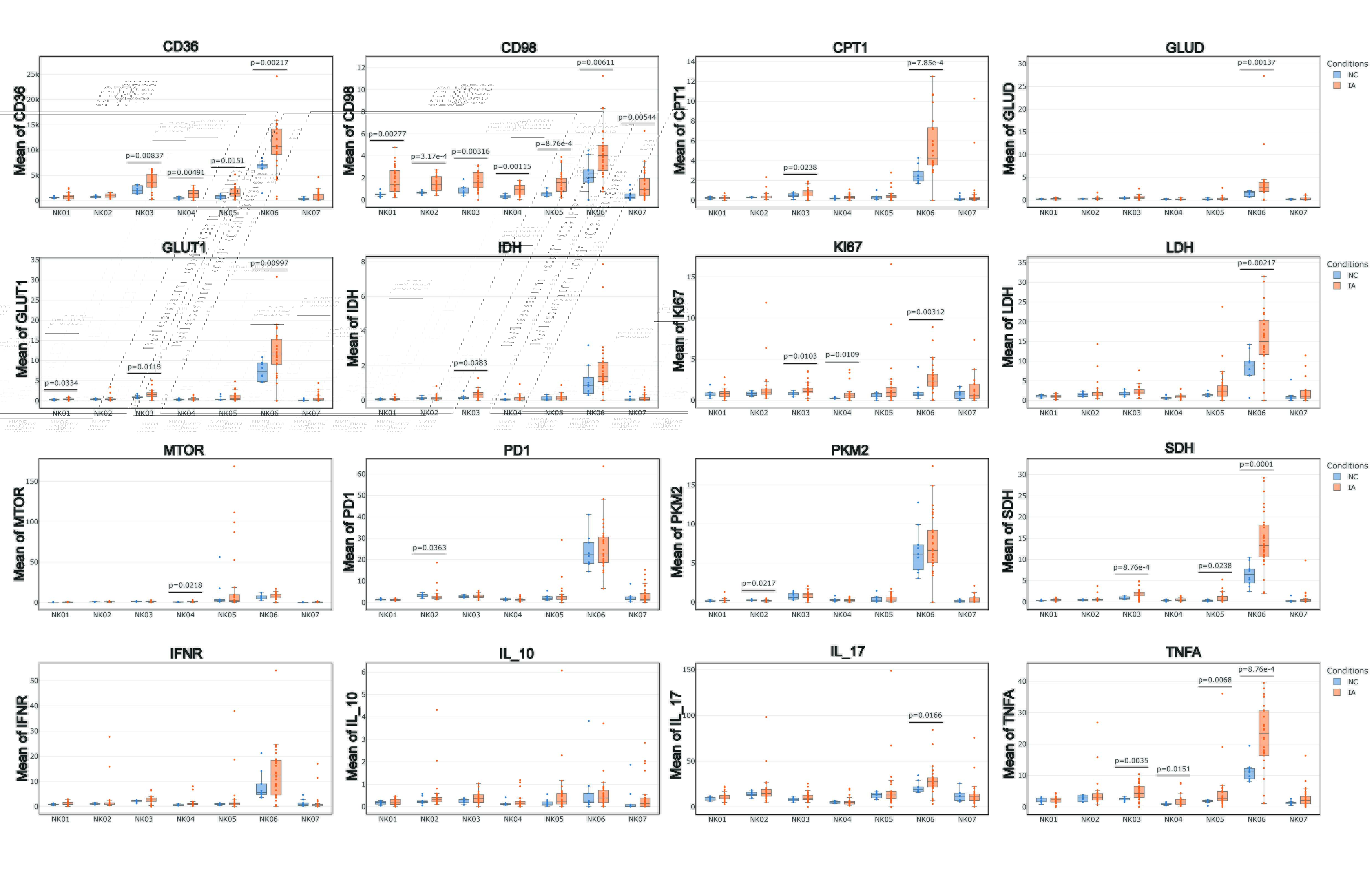


**Supplementary Figure 7:** Comprehensive comparison of functional and metabolic molecules among various NK subsets between NC and IA groups.


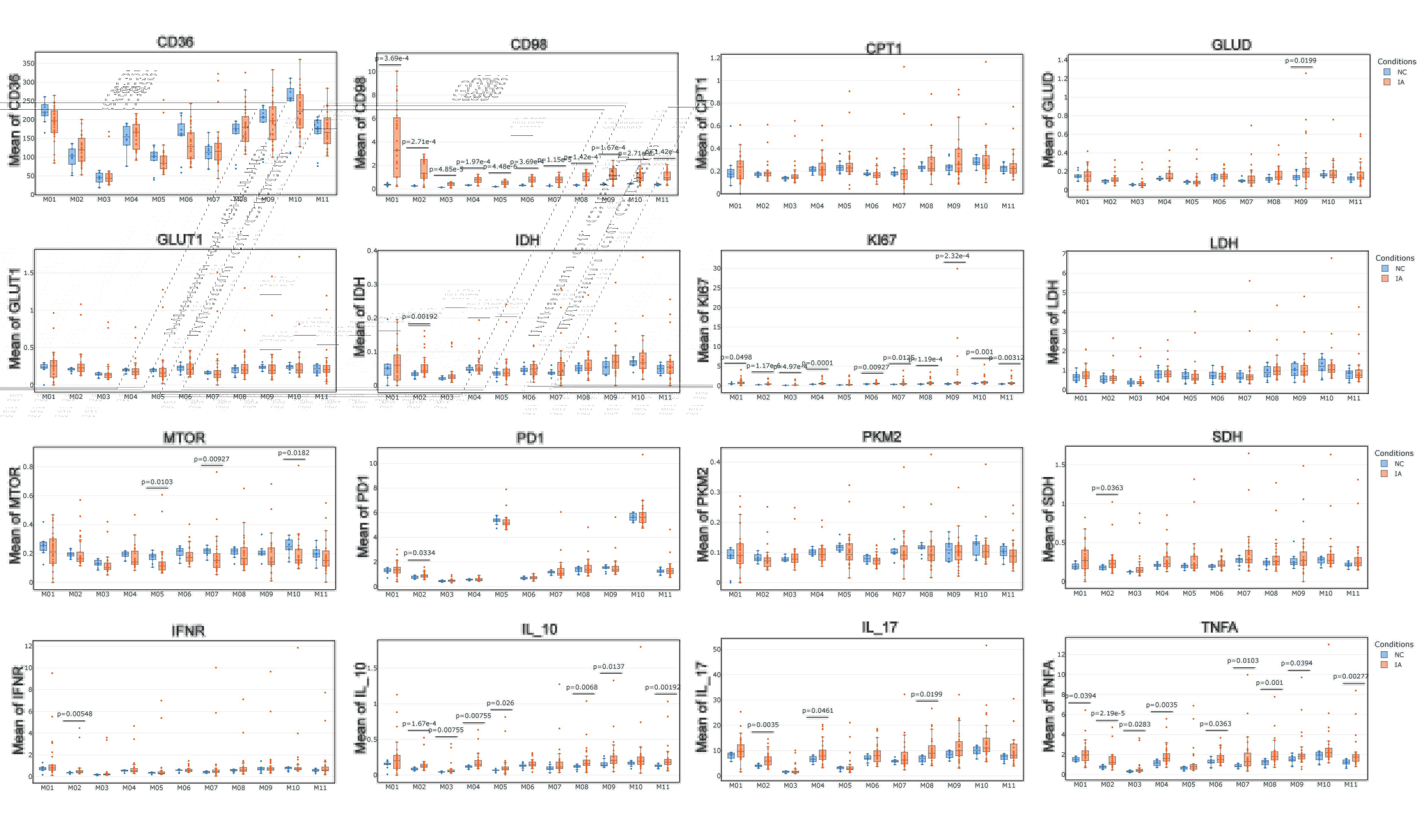


**Supplementary Figure 8:** Comprehensive comparison of functional and metabolic molecules among various monocyte subsets between NC and IA groups.


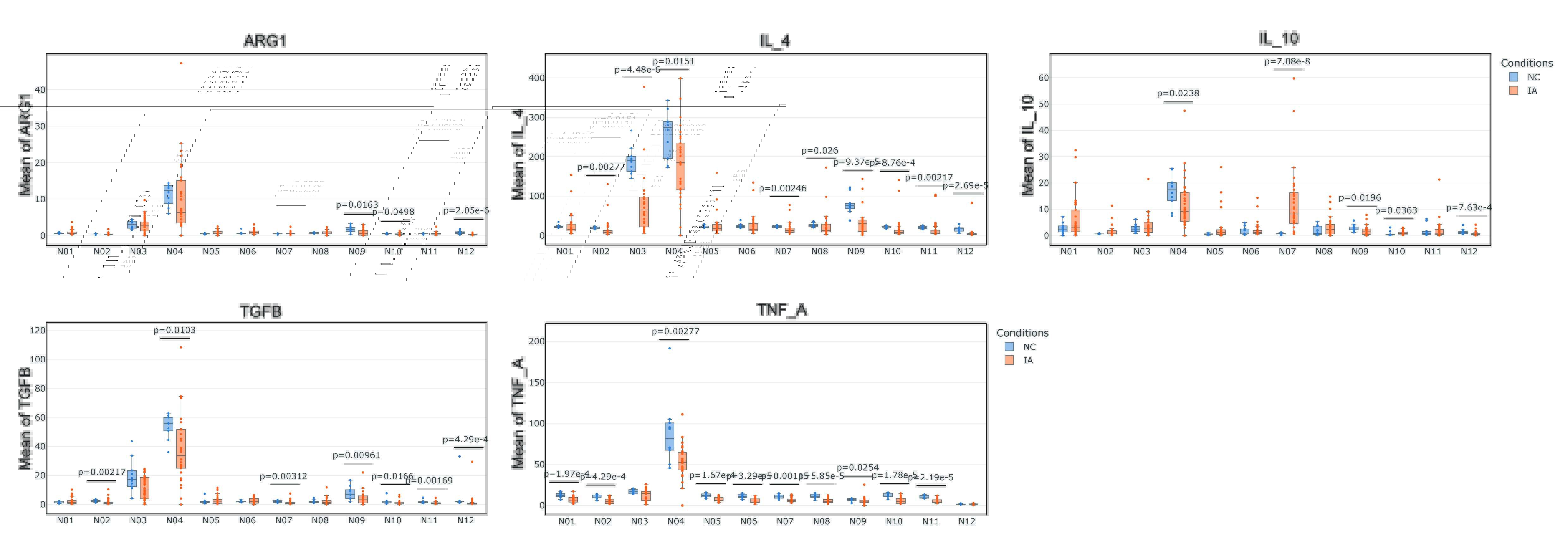


**Supplementary Figure 9:** Comprehensive comparison of functional molecules among various neutrophils subsets between NC and IA groups.
